# A constructional theory of sets, parts and relations

**DOI:** 10.1098/rsos.251390

**Published:** 2026-10-07

**Authors:** Salvatore Florio, Øystein Linnebo

**Affiliations:** University of Oslo, Oslo, Norway

**Keywords:** constructional ontology, set theory, mereology, plural logic, top-level ontology, knowledge representation

## Abstract

We develop a constructional approach to ontology, focusing on sets, parts and relations. On this conception of ontology, one starts with some constructors and an initial collection of objects. Then, new objects emerge by construction, that is, from the application of constructors to objects. This approach, which is inspired by work of Kit Fine, provides a unified formalization of core components of prominent formal ontologies. The resulting formal theory is also shaped by its intended application as a top-level ontology (TLO) for the Information Management Framework of the UK's National Digital Twin (NDT).

## 1. Introduction

The main aim of this article is to formulate a basic constructional ontology for three top-level categories: sets, parts and relations. In an ontology of this kind, an entity is accepted because it can be constructed from an initial stock of accepted objects—the *givens*—and constructors. The entire ontology unfolds from repeated constructions: it is generated through a process in which new entities are obtained by applications of constructors to available objects, which yield larger and larger domains of entities. So from the givens and constructors one can, in principle, know all the objects in the ontology.

After introducing the constructional approach to ontology (§2), we explain the benefits of the approach (§3) and develop a consistent formal theory to give it a rigourous formulation (§§4–6). Readers primarily interested in the general framework may want to focus on §§2–4. Sections 5 and 6 contain more technical material. To help readers less familiar with mathematical logic, we provide glosses of the axioms and informal explanations of the key ideas. The appendices describe various aspects of our approach, from the connection with other top-level ontologies (TLOs) to the consistency proof for the formal theory. The technical level varies depending on the topic.

Some features of the theory result from its intended application in the context of the UK's National Digital Twin (NDT). In the rest of the section, we elaborate on this context and on the initial motivation for the present work.

The National Infrastructure Commission's report ‘Data for the Public Good’ [1] recommended the creation of the UK digital twins across different sectors to give a system of systems view of national infrastructure [2]. Hetherington & West [3] suggested the adoption of an Information Management Framework that includes a Foundation Data Model (FDM) as a key component. It was proposed that the FDM be built on a TLO [3]. West [4] determined that four selected four-dimensionalist TLOs—the target TLOs—best met the technical requirements of the FDM: BORO, IDEAS, ISO 15926 and HQDM (Appendix C). It recommended that these target TLOs should be underpinned by rigorously established foundations. The theory developed in this article provides the rigourous foundations for these target TLOs.

Existing TLOs, including those selected as a starting point for the FDM, are typically assembled from disconnected core components. In the case of the target TLOs, such components correspond to sets, parts (mereological sums) and relations (tuples), which are not built and formalized in a single, unified way (see [5] for discussion and for a comprehensive survey of available TLOs). These components may be viewed as belonging to a single family and so should have similar formalizations [5, §4.2.1].

Previous work [6–9] has investigated how the selected TLOs could be given an integrated development based on the constructional framework put forward by Fine [10,11]. We provide a formalization of this novel approach, refactoring the disconnected core components into a unified theory. In keeping with terminology previously employed within the NDT programme, we call this unified ontology and its associated formal theory, respectively, the Core Constructional Ontology (CCO) and the Core Constructional Theory (CCT).

Our unified theory might be of interest beyond the selected TLOs. Other foundational theories make use of the core components refactored here—sets, parts and relations. They could thus benefit from a constructional development. One example is the Unified Foundational Ontology [12,13], which includes both sets and mereological sums.

## 2. Constructional ontology

In this section, we provide a brief overview of constructional ontology (see [14] for a more detailed exposition). As stated above, one starts with some constructors and some givens. Then, new objects emerge by construction, that is, from the application of constructors to objects. The resulting ontology can be characterized by three parameters: the givens with which one starts, the constructors one employs and the shape and extent of the constructional process. Among the objects that emerge from the applications of constructors, we allow both constructed objects—the direct outputs of the construction—and traces of the construction process itself. (We explain these ideas in later sections.)

Two features of constructional ontology are worth highlighting at the outset. First, it gives us a very clear picture of the overall contents of the ontology, including a comprehensive view of the categories of objects. For example, if the ontology includes only a set constructor, then all the objects in the ontology fall under one of these three kinds: givens, sets and traces of the construction process. We start with the givens. Then, sets and associated traces arise from repeated applications of the set constructor. In short, a constructor determines the category of the objects it generates.

Second, identity criteria emerge from the constructional process: the identity of constructed objects is dictated by their constructors and the inputs of the constructions. For example, two sets (i.e. objects obtained from the set constructor) are identical if and only if they are constructed from the same objects. In short, a constructor determines the identity of the objects it generates as well as their category.

Constructional ontology has a long and venerable history (e.g. [15–18]). Of special importance for our project is Kurt Gödel's articulation of ‘the concept of set […] according to which a set is anything obtainable from the integers (or some other well-defined objects) by iterated application of the operation ‘set of’ […]’, Gödel [19, p. 180]. Here, Gödel is referring to a broadly constructional concept of set: sets are generated by applications of the set constructor. More recent work on constructional ontology includes that of Kit Fine, who has significant influence on the present work. We build on a number of his ideas [10,11] for developing a unified constructional ontology.

## 3. Core Constructional Ontology

As we have mentioned, a constructional ontology can be characterized by three parameters: the givens with which one starts, the constructors one employs and the shape and extent of the constructional process. We begin to develop our approach, the CCO, by specifying these parameters.

The CCO assumes that the givens are the spatio-temporal atomic elements of all possible worlds—sometimes called the *pluriverse*. In other words, these atoms ‘cover’ the pluriverse. The assumption is compatible with different conceptions of the atoms. Moreover, the theory developed in this article, the CCT, does not constrain the choice of givens, which is left open. More specifically, the CCT only requires a minimal collection of givens: those that are necessary to characterize the construction process (see §6.3). This simplifies the task of formalization, where such a minimal collection is used. But the theory is consistent with stronger assumptions about the givens in the CCO and can be adapted straightforwardly to other settings.

Our approach involves a single, generic pattern for constructors, which we specialize into three key constructors: set, sum and ordered pair. This specialization involves characterizing the conditions under which these constructors can be applied. We work with ordered pairs because they are the simplest kind of tuples and, through nesting, can be used to represent other kinds of tuples.

From these emerge the key categories of constructed objects: sets, sums and ordered pairs. (Hereafter, we refer to ordered pairs simply as *pairs*, since unordered pairs do not play any important role in this context.) Note that, as new objects emerge, more possibilities for construction become available. For example, once two sets a and b have been constructed, we can construct their singletons or the set that has just these two objects as elements. We generate the target ontology by successively realizing every possible construction, based on the constructors that we have at our disposal.

In the remainder of this section, we focus on three features of our approach and explain their benefits for our project: the approach is foundational, it has a high degree of unification, and it is constructional in the sense introduced above.

Our ontology has three characteristics that enable it to play a foundational role. The first is its *categorical completeness*. That is, the ontology provides the three basic categories of objects for the target TLOs: sets, sums and tuples, together with their associated hierarchical relations (type-instance, super-sub-type, whole-part and tuple-place). The second characteristic is *object completeness*. That is, the ontology generates all the objects needed by the target TLOs. One might think of it as an ‘object factory’ which supplies the objects that might be needed in any domain. The third characteristic is that the basic categories provide objects in the ontology with appropriate *identity criteria*. These are broadly extensional, based on the type of constructor and its input. For example, two sets are identical if and only if they are constructed from the same objects. Similarly, two sums are identical if and only if they have the same parts.

Our approach is unified in the following ways. First, it gives a *common development of three key domains*: sets, sums and tuples. Ontologies that involve sets and sums usually adopt set theory and mereology as separate theories, without an integrated development. Here, we provide a unified treatment of sets, sums and pairs (hence tuples more generally) as *sui generis* objects. So the three target domains—sets, sums and tuples—arise in similar ways through construction. Second, there is a *common basis for identity criteria*, which are crucial for the foundational role discussed above. Identity criteria for the objects of the basic types are extensional, with differences arising from the way the objects are constructed. Third, the approach offers *uniform ways of capturing key commonalities and differences among objects of the basic types*. Such commonalities and differences are captured by features of the underlying constructors.

In the previous section, we outlined the basic structure of a constructional ontology. We now want to highlight four of its main benefits.

— *Categorical differences are constructional differences*: in constructional approaches, different kinds of objects can be distinguished from one another by the manner in which they are constructed. So categorical differences are explained by constructional differences.
— *Dependency*: some objects are built from others and hence ‘depend on’ them. This provides an explanation of ontological dependence.
— *Ontological economy*: the ontology is built out of a relatively small set of initial objects and thus achieves fundamental ontological economy in the sense of [20].
— *Consistency*: construction can be a basis for consistency, avoiding paradoxes such as those of Russell and Burali-Forti, although we need to take care with the construction process. In our case, this is achieved by requiring that the construction be ‘bottom-up’ in the sense that the properties of the constructed objects are determined by the properties of the inputs to the construction.

Gödel stresses this last benefit in the article cited above. Speaking of the constructional view of sets, he says:

[It] has never led to any antinomy whatsoever; that is, the perfectly ‘naïve’ and uncritical working with this concept of set has so far proved completely self-consistent. [19, p. 180]

We show that our approach can be developed consistently. We do so by providing a mathematical proof of consistency for the associated formalization.

Concerning dependency and ontological economy, we should note that the construction can be given a metaphysical interpretation as a form of grounding or non-eliminative reduction. However, we wish to remain neutral on the correct metaphysical interpretation (see [21,22] for more discussion).

## 4. Developing the Core Constructional Theory

In developing a formalization of the CCO, the first decision concerned what kind of constructional framework to adopt. We decided to go with the established, and thus safe, option of adopting a stage-theoretic framework (described below). This framework is well-developed for set theory but needed to be extended to sums and pairs. We also recognized that plurals have a natural role in representing the multiplicity of the input to our constructors, for at least two reasons. Plurals provide the most straightforward way of describing the multiplicity of inputs to construction. For example, it is far more natural to describe the set {a,b} as constructed from the *elements* a and b than from any single entity representing both a and b. This is how Gödel describes the ‘set of’ operation. And before Gödel, Georg Cantor famously defined a set in terms of a many-to-one operation: the ‘collection into a whole […] of determinate, well-distinguished objects’ [23, p. 481]. Moreover, alternative options—such as sets, classes, mereological sums and sequences—are unsuitable to serve as inputs. They cannot be presupposed by the construction process, since they are the very entities one might wish to construct. So a stage theory and plurals, regimented in plural logic, provided us with the overall framework for the CCT.

Our formalization takes its inspiration from the influential iterative conception of sets, which goes back to Gödel's article cited earlier: ‘What is Cantor's Continuum Problem?’ [19]. According to this conception, the sets are formed in stages. We start, at stage 0, with some urelements. At stage 1, we form all sets of urelements, which results in a larger domain. At stage 2, we form all sets of objects from this larger domain. We continue into the infinite. At limit stages, such as the first infinite stage ω, we collect the previously formed sets, which are then used to form new sets at the next stage ω+1. The most famous development of the iterative conception of sets is due to Boolos [24], who develops a stage theory—an axiomatic theory that describes how sets are successively formed at stages.

We generalize and extend Boolos's stage theory in three different ways. First, instead of considering just the construction (or ‘formation’) of sets, we adopt Fine's framework and offer a unified account of three different kinds of construction that are central to the target TLOs: sets, mereological sums and pairs. This enables us to capture the core hierarchical relations of the target TLOs mentioned earlier, namely, type-instance, super-sub-type, whole-part and tuple-place. We do not rely on a set-theoretic representation of pairs. Rather, we treat pairs as a category of constructed objects, distinct from that of sets. Our generic pattern for constructors relies on plural logic, which does not allow for order. So we reconstruct paired objects by means of special constructed objects marked by positions—we call them ‘position objects’. For pairs, we need two positions, so we introduce two categories of position objects. To emphasize that there is no assumed order, we call these ‘left objects’ and ‘right objects’ (we could have used a different naming convention, such as ‘red objects’ and ‘blue objects’). These objects are built by the corresponding constructors: the left constructor and the right constructor. For example, suppose that we want to construct the pair of a and b. We first construct from a and b two position objects $a^{\prime }$ and $b^{\prime }$, obtained, respectively, by applying the left constructor to a and the right constructor to b. Then, we apply the pair constructor to $a^{\prime }$ and $b^{\prime }$, which yields the desired pair.

Second, we do not require that all objects that can be constructed at a stage are constructed at that stage. This enables us to realize separately constructional possibilities that are independent of each other.

Third, the target ontologies involve *traces*, special objects that ‘log’ the structure of the construction process. These characterize how the objects are constructed. For example, suppose a set is constructed at a certain stage. Then, this stage also includes a trace for each element of the set constructed. These traces log the element's view of the construction: the element itself, the type of construction (set) and the output (the set constructed). For example, if the set constructed is {a,b}, there will be two related traces, one recording that a has been used as input in the construction of a set with output {a,b}, and the other recording that b has been similarly used. Thus, the overall construction of {a,b} is recorded by the plurality of these two traces. More details are provided in §6.14.

We describe the process of construction in stage-theoretic terms. The process consists of *stages* ordered by ⊴, which may be seen as an *accessibility relation* in the sense of relational semantics. We think of s⊴t quasi-temporally and therefore often pronounce it ‘s precedes t’ or ‘t is after s’. The accessibility relation is reflexive. So the intended sense of ‘s precedes t’ is ‘s precedes t or s is equal to t’. In our formalization, stages are neither givens nor constructed objects: they are part of the ‘infrastructure’ supporting the constructional process.

Every object in the target TLOs—sets, parts and tuples—is introduced at some stage. We write x@s for ‘x exists at s’. Some objects, the givens, exist at an initial stage. For pragmatic reasons, we suppose that there is a unique initial stage, and constructors will be the only givens whose existence is assumed (see §6.3). Once an object exists, it continues to exist at all later stages. However, as we move from earlier to later stages, new objects might be constructed and thus might begin to exist. In the investigation of the target TLOs, this process is called *ontogenesis* as, intuitively, after all the stages, all the objects in the ontology exist.

In describing the CCT, we tend to use a dynamic language. This makes the structure of construction easier to visualize, and it exposes relations of ontological dependence among objects (see §3). However, the resulting domain is not literally dynamic. It encompasses the objects that exist as a result of the entire process. The same is true of Boolos's stage theory for the iterative conception of set. While his theory describes a seemingly dynamic process of generation, the domain of the theory is not dynamic. It encompasses all sets and results from the entire process.

All construction is effected by means of constructors. A constructor is applied to appropriate objects that exist at some stage so as to construct another object, which may exist only at a later stage. In Appendix B, we provide details about the design choices that inform our approach.

To help non-specialist readers, we add informal glosses to the formal statements of the axioms. Moreover, where we make comments, we divide these into more accessible *notes* and technical *remarks*.

## 5. Core Constructional Theory: the formal language

In this section, we introduce the formal language in which the axioms of the CCT will be formulated (for the axioms, see §6). The language is given by the logical framework (plural logic) and some non-logical vocabulary. The non-logical vocabulary includes expressions for the key constructional operations and for auxiliary vocabulary needed to express the way in which the constructional operations are deployed through the stages.

### 5.1. Logical framework

As a logical framework for the CCT, we use plural logic, specifically what is known in the philosophical literature as PFO+, which is short for *plural first-order logic plus plural predicates*. This is the standard language for the formalization of plural logic, though there are some differences in notation among authors that use this system. Our exposition of the system relies on [25].

The language of PFO+ extends the language of first-order logic with the following classes of expressions:

— Plural terms: plural variables (vv,xx,yy,…, and variously indexed variants thereof) and plural constants (aa,bb,…, and variants thereof), roughly corresponding to the natural language pronoun ‘they’ and to plural proper names (e.g. ‘The Hebrides’), respectively.
— Universal and existential quantifiers that bind plural variables (e.g. ∀xx, ∃yy,…). We may read ‘∀xx’ as ‘whenever there are some things xx, then…’, and we may read ‘∃yy’ as ‘there are some things yy such that…’.
— A logical binary predicate ≺ for plural membership, corresponding to the natural language ‘is one of’ or ‘is among’. Its first argument place is reserved for singular terms, while the second is reserved for plural terms (e.g. ‘x≺xx’). So the predicate expresses a relation between an object and some objects. The intended reading of ‘x≺xx’ is ‘x is one of xx’, which may be pronounced ‘x is one of the xs’. For example, ‘s≺hh’ may be used to represent ‘Skye is one of the Hebrides’.
— Non-logical plural predicates, defined as predicates with one or more argument places reserved for plural terms. Like singular predicates, plural predicates have a fixed arity. Each argument place is exclusively singular or exclusively plural. That is, it is reserved exclusively for singular terms or exclusively for plural terms. An example is CONSTRUCT(z,xx,y), which represents that z is constructed from xx using the constructor type y. The predicate's arity can be represented by numerical superscripts, which may be omitted for economy, as in the preceding example. The full predicate is $\text{C}{\text{ONSTRUCT}}^{3}(z,xx,y)$. The first argument place is singular, and so is the third; the second argument place is plural.

The non-logical vocabulary of the language, which includes first-order and plural predicates (the fourth class identified above), is discussed in the next section.

We define a well-formed formula in the language of PFO+ starting with atomic formulae. These are formed by combining predicates with the appropriate number of terms. As noted earlier, we need to ensure that argument places reserved for terms of a certain kind (singular or plural) are occupied by terms of that kind. More complex formulae are obtained by means of sentential connectives and variable binding using singular or plural quantifiers. (See [25] for details.)

We should emphasize that plural predicates in this language are read collectively. Collective readings of plural predicates contrast with distributive readings. To appreciate the distinction, consider a natural language predicate such as ‘lifted a box’. The sentence ‘Annie and Bonnie lifted a box’ can mean that Annie and Bonnie lifted a box *together* (collective reading), or it can mean that Annie and Bonnie lifted a box *individually* (distributive reading). So a predicate in our formal language such as CONSTRUCT(z,xx,y) has a collective reading: it means that z is constructed collectively from xx using the constructor type y.

This choice of interpretation for the plural predicates does not make PFO+ any less expressive, since the effect of distributive predication can be obtained by paraphrase. For example, suppose we want to say that xx are sets, which have only a distributive reading, i.e. each of xx is a set. Then, using the singular predicate ISSET(x), we can express that xx are sets by saying that everything that is one of xx is a set:

∀x(x≺xx→ISSET(x)).

To illustrate the use of PFO+, it might be helpful to provide some examples of formulae together with informal glosses. For intuitiveness and simplicity, our informal glosses will often render plural expressions in terms of ‘pluralities’ and their members.

— v≺vv
  (v is one of vv, i.e. it is one of them.)
— ARESEVEN(aa)
  (aa are seven.)
— ISBOX(b)∧∃xx(∀x(x≺xx↔ISTILE(x)∧ISIN(x,b))∧WEIGH8KG(xx))
  (The tiles in box b weigh 8 kg together. Literally, b is a box and there are some things such that anything is one of them if and only if it is a tile in b and, together, they weigh 8 kg.)
— ∃z∃xx∃yCONSTRUCT(z,xx,y)
  (Some object is constructed from some things using some type of constructor.)
— ∃zz∀z(z≺zz↔(z=a∨z=b))
  (Consider two objects a and b, possibly identical. There is a plurality that has a and b, and only a and b, as its members. If a=b, then this is a ‘singleton’ plurality.)
— ∀z∀xx∀y(CONSTRUCT(z,xx,y)→∀ww(∀w(w≺xx↔w≺ww)→CONSTRUCT(z,ww,y)))
  (If an object of some type is constructed from a plurality xx, then it is also constructed by any plurality ww that has exactly the same members as xx.)

The language of PFO+ can be given a model-theoretic semantics within set theory, which will be crucial for the consistency proof (Appendix E). A model M consists of a set M serving as domain of quantification and an interpretation function i of terms and predicates. The first-order part of the language is interpreted in the usual way: the quantifiers range over M, monadic predicates are interpreted as sets of objects in M, relational predicates are interpreted as relations over M and singular predication is interpreted as set-theoretic membership. The plural part of the language is interpreted as follows. Plural quantifiers range over non-empty subsets of M. Plural membership is interpreted as set-theoretic membership. Monadic plural predicates are interpreted as sets of non-empty subsets of M. Relational plural predicates are interpreted as relations of objects in M and non-empty subsets of M, depending on whether each argument place is singular or plural. Plural predication is interpreted as set-theoretic membership. A detailed exposition of the semantics can be found in [25, §7.2].

The semantic clauses defining truth in a model extend those of first-order logic. For plural membership, we have that ‘a≺bb’ is true in a model M if and only if i(a)∈i(bb), that is, the interpretation of a is an element of the non-empty subset of M interpreting bb. The clauses for plural predication are best explained by means of examples. The monadic predication ‘WEIGH8KG(aa)’ is true in M if and only if i(aa)∈i(WEIGH8KG), that is, the non-empty subset of M interpreting aa is an element of the set of non-empty subsets of M interpreting WEIGH8KG. This also reflects the collective reading of plural predication: truth requires that the plurality itself be in the extension of the predicate. The relational predication ‘CONSTRUCT(a,bb,c)’ is true in M if and only if (i(a),i(bb),i(c))∈i(CONSTRUCT), that is, the triple of the interpretations of the arguments is an element of the relation interpreting CONSTRUCT.

### 5.2. Non-logical vocabulary

We have designed the language of the CCT so that its basic non-logical vocabulary can express the constructional operations in scope, the structure of the stages and the way in which operations and stages interact to produce the target ontology. This vocabulary is given by constants, first-order predicates and plural predicates of the fourth listed class in §5.1.

The present version of the CCT includes a single, generic constructor, which we express here as a three-place predicate rather than a function symbol. Given our requirements for formalization, this is the simpler choice. Using a function symbol for the generic constructor would require handling empty terms as well as partial functions, and therefore giving up classical logic. By expressing this constructor using a predicate, we retain classical logic and describe by means of axioms the conditions under which the relation of construction obtains between inputs and output (see §6.6). Different types of constructed object are obtained by switching one of the predicate's parameters. The switch is effected using special constants, singular terms representing each type of constructed objects.

**Table:** 

**generic constructor**	
*predicate*	*intended reading*
CONSTRUCT(z,xx,y)	z is an/the object constructed from xx using the type y

The predicate has three argument places. The first is associated to the object constructed, while the other two are associated to the inputs of the construction (a plurality of objects) and an object representing the type of construction effected.

To indicate the types of construction, we use six special constants.

**Table:** 

**types**	
*constant*	*intended reading*
$c_{\text{set}}$	set construction type
$c_{\text{sum}}$	sum construction type
$c_{\text{left}}$	left position construction type
$c_{\text{right}}$	right position construction type
$c_{\text{pair}}$	pair construction type
$c_{\text{union}}$	union construction type

For example, we describe the construction of a set by filling the third argument place of CONSTRUCT(z,xx,y) with the constant for the desired type of construction: $\text{C}\text{ONSTRUCT}(z,xx,c_{\text{set}})$.

Next, we have some predicates pertaining to stages and their structure.

**Table:** 

**stages**	
*predicate*	*intended reading*
STAGE(x)	x is a stage
⊴(s,t)	s is before, or equal to, t
@(x,s)	x exists at (stage) s

The process of construction involves the application of the generic constructor to some objects and the specification of the type of construction to be effected. Since we require pluralities to be non-empty (see §6), we thereby disallow empty inputs to any constructor. Moreover, one can refine this process by placing constraints—in accordance with the target TLOs—upon what objects can serve as inputs relative to particular types. In the case of the set constructor, for example, all objects other than position objects can serve as inputs and thus are (informally) ‘settable’. In the case of mereological sums, the CCT restricts the process of construction to *individuals*, namely, objects that are either givens or sums. Only individuals are ‘summable’. The notion of ‘individual’ is expressed by a primitive predicate.

**Table:** 

**individuals**	
*predicate*	*intended reading*
INDIVIDUAL(x)	x is either a given or a sum

As mentioned above, the target ontology involves traces, namely, objects that log the structure of the construction process. The components of this structure are accessed by three predicates listed below. More details about these predicates and their role are provided in §6.14.

**Table:** 

**traces**	
*predicate*	*intended reading*
HASOUTPUT(w,z)	w records a construction with output z
HASINPUT(w,x)	w records a construction with x as one of the inputs
HASTYPE(w,y)	w records a construction of type y

This completes our basic stock of primitive non-logical vocabulary. We shall make frequent use of definitions to introduce new notions. We treat these definitions as axioms rather than abbreviations. (This choice will also facilitate, in a subsequent project, the automated translation of the formalization into the Common Logic Interchange Format.) As a result, definitions introduce new primitive non-logical expressions relying on non-logical expressions from the basic stock, as well as prior definitions.

### 5.3. Conventions

To simplify the exposition of the CCT, we rely on a number of familiar conventions. Let us highlight some key examples.

First, we adopt the following formatting conventions.

— When parentheses are omitted, ambiguities should be resolved by applying the standard rules of precedence among logical connectives.
— Different styles of parentheses are used to make groupings of formulae easier to recognize.
— A leading block of universal quantifiers is frequently omitted. So axioms with free variables, i.e. variables not bound by a quantifier, are short for their universal closure.
— To improve readability, we use infix notation for some predicates. For example, we write ‘s⊴t’ rather than ‘⊴(s,t)’, and ‘x@t’ rather than ‘@(x,t).

Second, we adopt a convention related to restricted quantification.

— Quantification restricted to certain kinds of entities may be marked by the use of particular variables. We use s, $s^{\prime }$, $s_{0}$, t, etc. to range over stages. So ∀sφ(s) is short for ∀s(STAGE(s)→φ(s)). Similarly, we use ss, $ss^{\prime }$ andtt for plural quantification over stages. So ∀ssφ(ss) is short for ∀ss(∀x(x≺ss→STAGE(x))→φ(ss)).

Finally, we use the following, less common formatting convention in order to emphasize constructional links among argument places of relevant predicates.

— We use a colon rather than a comma to separate the argument place for the constructed object from the rest of the arguments. For example, we write CONSTRUCT(z:xx,y) rather than CONSTRUCT(z,xx,y) to indicate that z is constructed from xx and y.

## 6. Core Constructional Theory: the axioms

Our theory has several parts, with different concerns. The axiomatization aims to be perspicuous, user-friendly and modular rather than minimal. It is based on the language introduced in the preceding section.

### 6.1. Plural logic

The formal system PFO+, which we introduced in §5.1, comes equipped with logical axioms and rules of inference. The axioms and rules associated with the logical vocabulary of ordinary first-order logic are the standard ones. For example, one could rely on introduction and elimination rules for each logical expression. The plural quantifiers are governed by axioms or rules analogous to those governing the first-order quantifiers.

In addition, we have the following axioms or axiom schemes. First, every plurality is non-empty:

(A1) ∀xx∃yy≺xx

*(Every plurality has at least one member.)*

Then, there is an axiom scheme of indiscernibility:

(A2) ∀xx∀yy(∀z(z≺xx↔z≺yy)→(φ(xx)↔φ(yy)))

*(Coextensive pluralities satisfy the same formulae.)*

Some remarks are in order. First, the formula φ may contain parameters. So we have the universal closure of each instance of the displayed axiom scheme. Second, as is customary, we write φ(xx) for the result of replacing all free occurrences of some designated plural variable vv with ‘xx’ whenever ‘xx’ is substitutable for vv in φ (e.g. [26, 113]). Third, (A2) is a plural analogue of Leibniz's law of the indiscernibility of identicals, and as such, the scheme needs to be restricted to formulae φ(xx) that do not set up intensional contexts. Since there is no requirement for intensional contexts in the CCO, no such context will be introduced in the CCT. Thus, the axiom scheme may be used unrestrictedly in the present setting.

Finally, there is the unrestricted axiom scheme of *plural comprehension*, an intuitive principle that provides information about what pluralities there are. For any formula φ(x) in which x is free but xx is not, we have an axiom stating that if φ(x) is satisfied by at least one thing, then there are the things each of which satisfies φ(x):

(A3) ∃xφ(x)→∃xx∀x(x≺xx↔φ(x))

*(If something is φ, then there are some things that are all and only the φs. That is, if something is φ, then the φs exist.)*

We refer to an axiomatization of plural logic based on the principles just described as *full plural logic*. The fullness of the logic has to do with the fact that plural comprehension applies to any formula φ(x)—provided, as stated, that x but not xx occur free.

Two important relations in plural logic are introduced as definitions. One is the many–many relation of plural inclusion (‘are among’), which is symbolized as ‘≼’:

**Definition 1.**

xx≼yy↔∀z(z≺xx→z≺yy)

*(Some things xx are among yy when everything that is one of xx is one of yy.)*

Another important relation is plural identity, symbolized as ‘≈’. The relation can be defined using ‘≼’, the symbol introduced by the preceding definition:

**Definition 2.**

xx≈yy↔(xx≼yy∧yy≼xx)

*(Two pluralities xx and yy are identical if and only if xx are among yy and yy are among xx, i.e. xx and yy have the same members.)*

When deriving the axioms of set theory from the CCT (Appendix D.1), we assume another principle of plural logic, one that corresponds to the Axiom of Choice in set theory. Relying on pluralities of ordered pairs to code relations, the principle can be expressed in PFO+. Informally, it states that for any relation, there is a functional subrelation with the same domain. We do not adopt this plural principle as an official axiom of CCT. Rather, we leave it as an option available in contexts where the set-theoretic Axiom of Choice is needed.

### 6.2. Stages

The stages are linearly ordered by the accessibility relation ⊴:

(A4) ∀ss⊴s

*(⊴ is reflexive.)*

(A5) ∀s∀t(s⊴t∧t⊴s→s=t)

*(⊴ is antisymmetric.)*

(A6) $\forall s_{0}\forall s_{1}\forall s_{2}(s_{0}⊴s_{1}\;\wedge \;s_{1}⊴s_{2}\;\to s_{0}⊴s_{2})$

*(⊴ is transitive.)*

(A7) ∀s∀t(s⊴t∨t⊴s)

*(⊴ is connected.)*

In addition, stages are well-founded by ⊴. Let us define s⊲t (‘s is strictly before t’) as follows:

**Definition 3.**

s⊲t↔(s⊴t∧s≠t)

Then, we have:

(A8) ∀ss∃s(s≺ss∧¬∃t(t≺ss∧t⊲s))

*(The stages are well-founded by ⊴. That is, for any plurality ss of stages, there is a member s that is first among ss with respect to the order ⊴.)*

Notice that the last axiom makes essential use of plural logic, asserting something of every plurality of stages. Moreover, this axiom enables us to do well-founded induction on ⊴. (Assume that a property holds at the initial stage and that, whenever the property holds at every s such that s⊲t, then it also holds at t. Then the property holds at every stage.) Using this form of induction, we shall be able to show, for example, that, for every stage, something exists at that stage.

The accessibility relation is also serial:

(A9) ∀s∃ts⊲t

*(For every stage, there is a strictly later stage.)*

We say that *t is a successor stage of s* if and only if s precedes t and there is no stage strictly in between s and t, that is:

**Definition 4.**

SUCC(s,t)↔s⊲t∧¬∃u(s⊲u∧u⊲t)

Later, we introduce a related abbreviation, a predicate MAX(s,t) representing that t is a maximal extension of s, that is, that t contains the result of carrying out every construction that is possible at s (§6.15).

**Remark.**

*Axiom (A9) and the well-foundedness of ⊴ entail that every stage has a successor stage*.

**Remark.**

*We shall eventually see that the seriality of ⊴ can be proved from the other axioms of our theory (see §6.9). We keep the axiom of seriality, however, as we shall sometimes be interested in working with less than the full theory*.

There is an infinite stage:

(A10) ∃t(∃ss⊲t∧∀s(s⊲t→∃u(s⊲u∧u⊲t)))

*(There is a stage that is after some stage and is not immediately after any other stage.)*

**Note.**

*To see how this axiom secures an infinite stage, consider the finite stages* 0,1,2, … *and the first infinite stage* ω, *which comes after all the finite stages. To say that a stage* t *is not immediately after any other stage means that, if there is a stage* s *before* t, *then there is a stage between* s *and* t. *Both* 0 *and* ω *are not immediately after any other stage. (In the case of* 0, *this condition is vacuously satisfied). However, unlike* 0, ω *is after some stage. So we can characterize* ω *as the first stage that is after some stage but is not immediately after any other stage*.

**Remark.**

*We can now prove that there is an initial stage. Axiom (A10) entails that there is a stage. Thus, the plurality of stages exists and, given (A8), is well-founded. So there is a first stage*.

Let us say that some things exist at a stage if and only if each of them exists at that stage. So we define ‘xx@@s’ (‘xx exist at s’) as follows:

**Definition 5.**

xx@@s↔∀x(x≺xx→x@s)

We also assume a stage-theoretic version of the axiom scheme of replacement. This is a powerful principle, which provides information about how many stages there are. Specifically, the principle says there are so many stages that the objects available at any one of them do not suffice to reach arbitrarily high in the hierarchy of stages. That is, for any stage s, any objects xx at s, and any formula ψ(x,y) that represents a function from xx to objects throughout the hierarchy, there is a stage at which we find all the values that this function takes for arguments among xx.

(A11)

$$\begin{array}{rl} xx@@s\wedge \forall x(x≺xx\to \exists y & (\neg \text{S}\text{TAGE}(y)\wedge \forall z(\psi (x,z)↔y=z)))\;\to \\ & \exists t\forall x(x≺xx\to \forall y(\psi (x,y)\to y@t)) \end{array}$$

*(Suppose that* xx *exist at* s *and that* ψ(x,y) *represents a function mapping objects among* xx *to objects other than stages. Then, there is a stage* t *such that what exists at* t *includes the image under* ψ *of every member of* xx.)

This axiom is included primarily for the sake of re-construing set theory; it is not essential to our approach. Notice, though, that our replacement axiom is not about sets but about stages and the many objects that exist at various stages. Indeed, replacement is the first of our axioms that ties the existence of stages to the existence of objects that exist at stages, i.e. it is the first ‘interactive’ axiom.

### 6.3. Initial stage

An initial stage is one that is not preceded by any other stage. Let us define ‘INIT(s)’ to mean that s is initial:

**Definition 6.**

INIT(s)↔¬∃tt⊲s

**Remark.**

*It follows from the connectedness of* ⊴ *that* s *is initial if and only if it is before every other stage, i.e*. ∀ts⊴t. *This also means that there is at most one initial stage. In a different context, the current definition of initial stage would permit several alternative initial stages*.

We define an object to be a given if and only if it exists at some initial stage:

**Definition 7.**

GIVEN(x)↔∃s(INIT(s)∧x@s)

Then, we assume the existence of some given:

(A12) ∃xGIVEN(x)

*(There is some given.)*

In our setting, we need objects that code information about types of construction. So we assume the existence of such objects. For example, we introduce an object $c_{\text{set}}$ that stands for the type *set*. We do the same for each type of construction in scope: *sum*, *left object*, *right object*, *pair* and *union*. We make the pragmatic choice to treat these objects as givens and, in fact, as the only givens whose existence is assumed. In the future, we might want to separate these objects from those that more properly represent the intended constructional ontology. For this specific implementation of the CCT, and to allow modularity, we have the following strengthening of (A12):

(A13) $\text{G}\text{IVEN}(c_{\text{set}})\;\wedge \;\text{G}\text{IVEN}(c_{\text{sum}})\;\wedge \;\text{G}\text{IVEN}(c_{\text{left}})\;\wedge \;\text{G}\text{IVEN}(c_{\text{right}})\;\wedge \;\text{G}\text{IVEN}(c_{\text{pair}})\;\wedge \;\text{G}\text{IVEN}(c_{\text{union}})$

*(The givens include:* $c_{\text{set}}$, $c_{\text{sum}}$, $c_{\text{left}}$, $c_{\text{right}}$, $c_{\text{pair}}$ $\;\text{and}\;c_{\text{union}}$.*)*

These are six distinct givens:

(A14) $c_{\text{set}}\neq c_{\text{sum}}\;\wedge \;c_{\text{set}}\neq c_{\text{left}}\;\wedge \;c_{\text{set}}\neq c_{\text{right}}\;\wedge \;c_{\text{set}}\neq c_{\text{pair}}\;\wedge \;c_{\text{set}}\neq c_{\text{union}}\;\wedge \;c_{\text{sum}}\neq c_{\text{left}}\;\wedge \;c_{\text{sum}}\neq c_{\text{right}}\;\wedge \;c_{\text{sum}}\neq$ $c_{\text{pair}}\;\wedge \;c_{\text{sum}}\neq c_{\text{union}}\;\wedge \;c_{\text{left}}\neq c_{\text{right}}\;\wedge \;c_{\text{left}}\neq c_{\text{pair}}\;\wedge \;c_{\text{left}}\neq c_{\text{union}}\;\wedge \;c_{\text{right}}\neq c_{\text{pair}}\;\wedge \;c_{\text{right}}\neq c_{\text{union}}\;\wedge \;c_{\text{pair}}\neq c_{\text{union}}$

It is convenient to have a separate predicate applying to these objects *qua* representations of types of construction:

**Definition 8.**

$$\begin{array}{rl} \text{T}\text{YPE}(y)\;↔\; & (y=c_{\text{set}}\;\vee \;y=c_{\text{sum}}\;\vee \;y=c_{\text{left}}\;\vee \\ & \;y=c_{\text{right}}\;\vee \;y=c_{\text{pair}}\;\vee \;y=c_{\text{union}}) \end{array}$$

In §6.13, we shall see that the type union has a *derived* status, while the remaining types are *basic*.

**Remark.**

*Our approach can be extended to other constructors one may wish to add. If new constructors are adopted, then the definition of* TYPE(y) *will have to be modified accordingly. However, as we shall see, other axioms that use this predicate can remain unchanged, thus allowing a pleasing modularity*.

### 6.4. What exists at stages

Some of the previous axioms already provide information about what exists at stages. For example, stipulating the existence of some givens implies that they exist at the initial stage. Moreover, the axiom scheme of replacement informs us that if some objects exist at a stage, their images under a functional relation also exist at some stage—provided only that no image is a stage. It is worth emphasizing that while stages are part of the CCT, they are not part of the CCO. We use the term ‘object’ in two corresponding ways. In a wide sense, it stands for all entities in the domain of the CCT. In a narrow sense, it stands for relevant entities in the CCO and thus excludes stages. (So far we have used the term primarily in the narrow sense.) We now continue to explore principles that connect stages and what exists.

First, we have an axiom that ties the objects in our ontology to stages, with the exception of the stages themselves, which are not part of the intended ontology and thus have a purely auxiliary role. (In future work, we shall explore formalizations that do rely on stages as primitive.)

(A15)  ∀x(¬STAGE(x)→∃sx@s)

*(Everything that is not a stage exists at some stage.)*

**Note.**

*We shall be able to prove the other direction of the conditional in (A15) relying also on axioms introduced in later sections. So, in the CCT, something is not a stage if and only if it exists at some stage*.

We identify stages extensionally. That is, stages with identical domains are identical:

(A16)  ∀x(x@s↔x@t)→s=t

*(Stages with identical domains are identical.)*

Moreover, stages are ‘cumulative’:

(A17)  s⊴t∧x@s→x@t

*(When one stage precedes another, then everything that exists at the former also exists at the latter.)*

Define a stage t to be the least upper bound (LUB) of some stages ss in the usual way:

**Definition 9.**

$\text{LUB}(t,ss)↔\forall s(s≺ss\to s⊴t)\wedge \forall t^{\prime }(\forall s(s≺ss\to s⊴t^{\prime })\to t⊴t^{\prime })$

*(According to the definition*, t *is the LUB of* ss *if and only if two conditions are met: (i)* t *is an upper bound of* ss, *i.e*. t *is after, or equal to, any stage in* ss, *and (ii)* t *is the least among the upper bounds of* ss, *i.e*. t *is before, or equal to, any upper bound of* ss.*)*

Next, we have an axiom characterizing ‘collection’ stages. No new object is introduced at these stages; they merely collect objects existing at previous stages. Collection stages are useful when, as in our case, the construction process is extended into the transfinite and hence requires infinite stages. To see why, consider the first infinite stage ω, which comes after all finite stages. We cannot think of ω as introducing new objects constructed from those available at the preceding stage. This is because there is no stage that immediately precedes ω: any stage n prior to ω has a successor stage n+1 that is also prior to ω. So what happens at stage ω? We adopt the option of simply letting ω collect the objects that exist at prior stages. The same applies to other infinite stages that are limit, namely, neither an initial stage nor a successor one. The following entails that limit stages are collection stages (and it provides trivial information about successor stages):

(A18) LUB(t,ss)→∀x(x@t→∃s(s≺ss∧x@s))

*(Suppose t is the LUB of some stages ss. Then, everything that exists at t exists at some of ss.)*

**Remark.**

*Since stages are cumulative, we can strengthen the axiom's consequent to the claim that, for every* x, x *exists at* t *if and only if* x *exists at some of* ss.

We now want to define a predicate CONSTRFROM(x,s) whose intuitive meaning is that x is constructed from some objects that exist at stage s. The precise definition is as follows:

**Definition 10.**

CONSTRFROM(x,s)↔∃xx∃y(xx@@s∧TYPE(y)∧CONSTRUCT(x:xx,y))

Finally, we restrict what can exist at a successor stage. This axiom also uses the notion of trace, which is characterized in §6.14.

(A19) SUCC(s,t)∧x@t→x@s∨CONSTRFROM(x,s)∨TRACE(x)

*(Everything that exists at a successor stage either existed at the predecessor stage, or is constructed from something at that stage, or is a trace.)*

Subsequent axioms will ensure the existence of appropriate traces at each successor stage. In particular, they will ensure that new traces emerging at a successor stage correspond to constructions effected at that stage.

### 6.5. What is and is not constructible

We impose various constraints on which objects can serve as inputs to different types of construction. These constraints do not appear in the framework of Fine [11]. They are added here to reflect the constraints in the target TLOs and could easily be lifted in connection with the pursuit of other goals. In this section, we provide an informal characterization of the constraints. Formal renderings of them will be developed in the later sections, where each type of construction is axiomatized.

Let us recapitulate the overall structure of our ontology. We have five basic types of objects that can be constructed: set, sum, two types of position objects (left object and right object) and pair. One additional type of object is derived: union. The construction process proceeds in stages, beginning with some givens. It yields objects of basic and derived types as well as traces storing information about the constructions effected.

The construction of sets is the least constrained operation. Sets, sums, pairs, givens and traces can all serve as inputs to constructions of sets or, as we may put it, are ‘settable’. Moreover, any plurality mixing objects of settable types is itself settable. By contrast, the construction of sums is the most constrained operation. Only individuals can serve as inputs, where an individual is defined as follows:

**Definition 11.**

$\text{I}\text{NDIVIDUAL}(x)↔(\text{G}\text{IVEN}(x)\;\vee \;\exists xx\;\text{C}\text{ONSTRUCT}(x:xx,c_{\text{sum}}))$

*(Any object is an individual if and only if it is either a given or a sum. )*

**Remark.**

*The axioms of the CCT entail that sets, position objects and pairs are not individuals. More generally, any two objects constructed from distinct types other than union are distinct. Unions are sets. (See §6.8.)*

Position objects resemble sets in the following respect: the objects that can be used as inputs for their construction are exactly sets, individuals (i.e. givens and sums), pairs and traces. So position objects are not themselves ‘positionable’. There is a difference, however. The input to the construction of a position object must be a ‘singleton plurality’, namely, a plurality with only one member. So any set, sum, pair or trace can be the single member of a plurality eligible for the construction of a left or right object.

Pairs can be constructed from position objects and only from them. But the configuration of the input plurality must respect two conditions: (i) the plurality contains exactly two members, (ii) one member is a left object while the other member is a right object.

To sum up, the constraints vary according to the type of construction. The difference might concern the type of objects in the input plurality or the size of that input.

### 6.6. Generic constructor

The generic constructor is functional in the following sense:

(A20)

$$\begin{array}{rl} & \text{C}\text{ONSTRUCT}(z_{1}:xx_{1},y_{1})\;\wedge \;\text{C}\text{ONSTRUCT}(z_{2}:xx_{2},y_{2})\;\wedge \;\\ & \;y_{1}=y_{2}\;\wedge \;xx_{1}\approx xx_{2}\;\to \;z_{1}=z_{2} \end{array}$$

*(Any two objects of the same type, constructed from the same pluralities, are identical.)*

We also require that the third argument place be a type.

(A21) CONSTRUCT(z:xx,y)→TYPE(y)

*(If an object z is constructed from xx using y, then y is a type.)*

### 6.7. Specialized constructors

We need axioms that characterize the behaviour of the four basic types of construction, including their domains of application, and fix the identity criteria of their outputs. This is done in the next four sections, which cover, respectively, sets, sums, position objects and pairs.

For ease of exposition, it is useful to define operations that represent the construction of objects of each type.

**Definition 12.**

$\text{S}\text{ET}(x:xx)↔\text{C}\text{ONSTRUCT}(x:xx,c_{\text{set}})$

**Definition 13.**

$\text{S}\text{UM}(x:xx)↔\text{C}\text{ONSTRUCT}(x:xx,c_{\text{sum}})$

**Definition 14.**

$\text{L}\text{EFT}(x:xx)↔\text{C}\text{ONSTRUCT}(x:xx,c_{\text{left}})$

**Definition 15.**

$\text{R}\text{IGHT}(x:xx)↔\text{C}\text{ONSTRUCT}(x:xx,c_{\text{right}})$

**Definition 16.**

$\text{P}\text{AIR}(x:xx)↔\text{C}\text{ONSTRUCT}(x:xx,c_{\text{pair}})$

**Definition 17.**

$\text{U}\text{NION}(x:xx)↔\text{C}\text{ONSTRUCT}(x:xx,c_{\text{union}})$

It is then convenient to speak as if we are dealing with different types of constructor. For example, we refer to the ‘set constructor’ and describe its properties. However, it is important to keep in mind that the CCT is built around a single, generic constructor that can be specialized to obtain different types of objects. So the notion of ‘set constructor’ is ultimately understood in terms of applications of the generic constructor using the parameter $c_{\text{set}}$.

We use the following definitions to capture the extension of each type of constructed object.

**Definition 18.**

ISSET(x)↔∃xxSET(x:xx)

**Definition 19.**

ISSUM(x)↔∃xxSUM(x:xx)

**Definition 20.**

ISLEFT(x)↔∃xxLEFT(x:xx)

**Definition 21.**

ISRIGHT(x)↔∃xxRIGHT(x:xx)

**Definition 22.**

ISPAIR(x)↔∃xxPAIR(x:xx)

**Definition 23.**

ISUNION(x)↔∃xxUNION(x:xx)

We wish to highlight an important feature possessed by some but not all types of constructed objects. Unlike sums, sets, positions objects and pairs are strictly dependent on their members: if two objects of the relevant type are identical, they are constructed from the same plurality. In other words, an object of the relevant type is constructed from at most one plurality. This feature corresponds to the *injectivity* of the relevant type, which we express as follows:

**Definition 24.**

$$\begin{array}{rl} & \text{I}\text{NJECTIVE}(y)\;↔\;\text{T}\text{YPE}(y)\;\wedge \;\\ & \;\forall z_{1}\forall xx_{1}\forall z_{2}\forall xx_{2}(\text{C}\text{ONSTRUCT}(z_{1}:xx_{1},y)\;\wedge \;\text{C}\text{ONSTRUCT}(z_{2}:xx_{2},y)\;\to \;\\ & \;\;\;\;\;\;\;(z_{1}=z_{2}\;\to \;xx_{1}\approx xx_{2})) \end{array}$$

### 6.8. Classification

We want to classify the objects in the domain of the CCT. Our target principles are:

— everything is either an individual, a set, a left object, a right object, a pair, a trace or a stage;
— nothing has more than one of the mentioned properties.

The first principle can be obtained from the preceding axioms. This can be seen as follows. By the axioms concerning what exists at a stage, we have that anything that is not a stage exists at some stage. The only things that exist at stages are givens, constructed objects and traces. Givens are individuals, and a constructed object is a set, a sum (thus an individual), a position object or a pair. A union is a set and thus does not introduce an extra dimension in the classification.

The second principle is assumed as an axiom.

(A22)

$$\begin{array}{rl} & \forall z_{1}\forall z_{2}\forall xx_{1}\forall xx_{2}\forall y_{1}\forall y_{2}(\text{C}\text{ONSTRUCT}(z_{1}:xx_{1},y_{1})\wedge \text{C}\text{ONSTRUCT}(z_{2}:xx_{2},y_{2})\\ & \;\;\;\;\wedge y_{1}\neq y_{2}\wedge y_{1}\neq c_{\text{union}}\wedge y_{2}\neq c_{\text{union}}\to z_{1}\neq z_{2})\;\wedge \\ & \forall z(\exists xx\exists y\text{C}\text{ONSTRUCT}(z:xx,y)\to \neg \text{T}\text{RACE}(z)\wedge \neg \text{S}\text{TAGE}(z))\;\wedge \\ & \forall z(\text{T}\text{RACE}(z)\to \neg \text{S}\text{TAGE}(z)) \end{array}$$

*(Nothing has more than one of the relevant properties. More explicitly, any two objects constructed from distinct types other than union are distinct; constructed objects are distinct from traces and stages; and traces are distinct from stages.)*

**Note.**

*The type of the union constructor must be excluded since unions are sets. However, the characterization of unions (§6.13) ensures that they identified with sets*.

### 6.9. Set constructor

We begin by defining the permissible inputs to the set constructor, as anticipated in §6.5:

Definition 25.
$$\begin{array}{rl} & \text{S}\text{ETTABLE}(x)\;↔\;\\ & \;(\text{I}\text{S}\text{S}\text{ET}(x)\;\vee \;\text{I}\text{NDIVIDUAL}(x)\vee \;\text{I}\text{S}\text{P}\text{AIR}(x)\;\vee \;\text{T}\text{RACE}(x)) \end{array}$$

We use the definition to constrain the application of the set constructor:

(A23) ∀xx∀x(SET(x:xx)→∀z(z≺xx→SETTABLE(z)))

*(If some things construct a set, then each of them is settable.)*

The next axioms specify the relation between sets, their members and stages. We require that every appropriate plurality existing at a stage is eventually used to construct a set:

(A24)

$$\begin{array}{rl} & \forall xx\forall s(xx@@s\;\wedge \forall x(x≺xx\;\to \;\text{S}\text{ETTABLE}(x))\;\to \;\\ & \;\;\exists x\text{S}\text{ET}(x:xx)) \end{array}$$

*(For every plurality xx of settable objects existing at s, the set of xx exists.)*

Analogous requirements will apply to other constructors. We also lay down that sets strictly depend on their elements:

(A25) $\text{I}\text{NJECTIVE}(c_{\text{set}})$

*(The type set is injective. That is, a set is constructed from at most one plurality.)*

**Remark.**

*The axioms of the CCT entail that the elements of a set exist at an earlier stage than the set itself:*

∀x∀xx(x@t∧SET(x:xx)→∃s(s⊲t∧xx@@s)).

*We reason as follows. Suppose that a set x exists at a stage. Because limit stages are collection stages, x must exist at the initial stage or at a successor stage. It cannot exist at the initial stage, since the givens are not sets. So x must exist at a successor stage. Let t be the least successor stage at which x exists. Since x does not exist prior to t and it is not a trace, (A19) yields that the elements of x exist at a stage earlier than t. This reasoning is based on previous axioms, including the fact that the basic kinds of objects (sets, individuals, position objects, pairs and traces) do not overlap, a fact sanctioned by the classification axiom (A22)*.

In this setting, there is a natural definition of set-theoretic membership:

**Definition 26.**

x∈y↔∃yy(SET(y:yy)∧x≺yy)

That is, to be an element of a set is to be a member of the (unique) input plurality from which the set is constructed.

**Remark.**

*Every set is constructed from some plurality and thus has the members of that plurality as elements. Since pluralities are not empty, it follows that there is no empty set in this setting. Moreover, the functionality of the generic constructor and the injectivity of the set constructor yield an analogue of the usual extensionality criterion for set: two sets are identical if and only if they are constructed from the same pluarlity. In symbols:*

SET(x:xx)∧SET(y:yy)→(xx≈yy↔x=y)

**Remark.**

*Fine [11] discusses four key principles for the identity of constructed entities: collapse (C), levelling (L), absorption (A) and permutation (P). Here, we simply note that the preceding axioms enable us to derive the correct CLAP profile for sets, namely*, 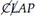.

**Remark.**

*Given the set constructor and the axioms governing it, it follows that the stages are serial, that is, for every stage, there is a strictly later stage: ∀s∃ts⊲t. The argument exploits a version of Russell's paradox. Consider a stage s. Use plural comprehension to consider the plurality xx of all and only those objects at s that are not elements of themselves. This plurality can be used to construct a set y at a stage t, with s⊴t. We want to show that t is strictly after s: s⊲t. Suppose not, i.e. that t=s. We now ask whether y∈y, with ∈ defined by definition 26. Since y has as its elements all and only the objects at s that are not elements of themselves, and since y by assumption exists at s, it follows that y is an element of itself if and only if it is not an element of itself. Since this is a contradiction, we conclude that s≠t and thus that s⊲t after all, as desired*.

In Appendix D.1, we state the axioms of Zermelo–Fraenkel set theory with the Axiom of Choice (ZFC), modified to fit the present context. We then derive such axioms from those of the CCT (Appendix D.1). To facilitate the derivation, we adopt an alternative, set-theoretic version of the axiom that there is an infinite stage (§6.2):

(A26)

$$\begin{array}{rl} & \exists xx\exists s\exists y(xx@@s\;\wedge \;\text{G}\text{IVEN}(y)\;\wedge \;y≺xx\;\wedge \;\forall x(x≺xx\;\to \\ & \;\;\;\exists u\exists uu(\text{S}\text{ET}(u:uu)\;\wedge \;\forall w(w≺uu\;↔\;w=x)\;\wedge \;u≺xx))) \end{array}$$

*(There are some xx such that xx exist at a stage, xx have a given as a member and, whenever x is a member of xx, its singleton {x} is also a member of xx.)*

In the context of our theory, this axiom is equivalent to our earlier assumption that there is an infinite stage. For the plurality required by (A26) to exist can only arise at a limit stage. Therefore, once this axiom is adopted, we could dispense with our earlier assumption.

### 6.10. Sum constructor

The inputs of the sum constructor are constrained to include only individuals:

**Definition 27.**

SUMMABLE(x)↔INDIVIDUAL(x)

We make the corresponding requirement:

(A27) ∀xx∀x(SUM(x:xx)→∀z(z≺xx→SUMMABLE(z)))

*(If some things construct a sum, then each of them is summable.)*

**Remark.**

*It follows from our assumptions that all givens are summable*.

As with sets and other types of constructed objects, we require that every appropriate plurality existing at a stage is eventually used to construct a sum:

(A28) ∀z(z≺xx→SUMMABLE(z)∧z@s)→∃xSUM(x:xx)

*(For every plurality xx of summable objects existing at a stage s, the sum of xx exists.)*

**Note.**

*In the case of sets, we have that the elements of any set exist at a stage strictly before any stage at which the set itself exists. Does something analogous hold of sums?*

*The answer is negative: sums are simply different from sets in this regard. To see this, consider a given g and let gg be the plurality whose sole member is g. Then, as we shall see shortly, SUM(g:gg). This provides a counter example to the hypothesis that every sum is the sum of some objects that exist strictly before the sum itself*.

Next, we need to state a criterion of identity for sums. Before we do that, however, we state two constraints on their individuation, which Fine [11] labels *collapse* and *levelling*. First, sums satisfy collapse:

(A29) SUM(x:xx)∧∀u(u≺xx↔u=y)→x=y

*(The sum constructed from the singleton plurality of x is x.)*

Second, sums satisfy levelling:

(A30)

$$\begin{array}{rl} \text{S}\text{UM} & (x:xx)\;\wedge \;\text{S}\text{UM}(y:yy)\;\wedge \\ \forall & z_{1}(z_{1}≺xx\;\to \;(z_{1}≺yy\;\vee \exists zz(zz≼yy\;\wedge \;\text{S}\text{UM}(z_{1}:zz))))\;\wedge \\ \forall & z_{2}(z_{2}≺yy\;\to \;(z_{2}≺xx\;\vee \\ & \;\exists zz(zz≼yy\;\wedge \;z_{2}≺zz\;\wedge \;\exists z_{3}(z_{3}≺xx\;\wedge \;\text{S}\text{UM}(z_{3}:zz)))))\;\to \\ & \;\;\;x=y \end{array}$$

*(Suppose x is a sum of xx and y is a sum of yy. If the following two conditions are satisfied, then x is identical with y:*

— *every x among xx is either one of yy or is a sum of some zz among yy;*
— *every y among yy is either one of xx or is one of some members of yy whose sum is among* xx.

*In other words, two pluralities, one obtained from the other by replacing some pluralities of objects with their respective sums, yield the same sum.)*

**Remark.**

*Given the axioms of plural logic, the functionality of the generic constructor, (A20), entails absorption and permutation for any type of constructor. Thus, (A20), (A29) and (A30) lay down the entire CLAP profile of sums, namely, full CLAP*.

Axioms (A20), (A29) and (A30) provide *sufficient* conditions for two mereological sums to be identical. But we do not yet have any *necessary* conditions. Let us explore the possibility of adding an ‘extremal clause’ to the effect that no two sums are identical unless they are identified as a result of these three axioms. The three axioms can then be regarded as a recursive definition of sum identity. Our question, then, is how this extremal clause might be formulated.

We first observe that we can prove that *every sum is a sum of givens*. The proof goes by induction on the construction of sum (see §6.16 for a general version of induction on construction). Let us sketch the reasoning, which relies essentially on the fact that givens and sums are individuals and that only individuals are summable. First, by collapse, every given is a sum of givens. Second, assuming that each of some summable objects xx is a sum of givens, it follows by levelling that so too is the sum of xx. Thus, the promised conclusion follows by induction on stages.

This observation means that, to provide a criterion of identity for sums, it suffices to state a criterion for sums of givens. In fact, it turns out there is a simple way to do so, that is, to adopt the ‘hyperextensional’ principle of Goodman [18]:

(A31)

$$\begin{array}{rl} & \forall x_{1}(x_{1}≺xx_{1}\to \text{G}\text{IVEN}(x_{1}))\;\wedge \;\forall x_{2}(x_{2}≺xx_{2}\to \text{G}\text{IVEN}(x_{2}))\;\wedge \;\\ & \;\;\text{S}\text{UM}(z_{2}:xx_{1})\;\wedge \;\text{S}\text{UM}(z_{2}:xx_{2})\to \\ & \;\;\;\;\;\;\;(z_{1}=z_{2}\;\to \;xx_{1}\approx xx_{2}) \end{array}$$

*(If two sums of givens are identical, those givens must also be identical. That is, a sum can be constructed from givens $xx_{1}$ and also from givens $xx_{2}$, then $xx_{1}$ are the very same objects as $xx_{2}$.)*

This is pleasingly analogous to the strict dependence that holds for sets, captured by the injectivity of the set constructor. Since every sum is a sum of givens, every sum is constructed from a unique plurality of givens. So we have an extensional criterion of identity for sums: two sums are identical if and only if they are constructed from the same givens. In other words, the sum constructor is injective on pluralities of givens.

We adopt the following definition of a predicate ≤, where the intended meaning of ‘x≤y’ is that x is a (mereological) part of y:

**Definition 28.**

x≤y↔∃yy(SUM(y:yy)∧x≺yy)

So to be a part of an object is to be a member of a plurality from which the object can be built using the sum constructor. This definition is analogous to the definition of set-theoretic membership (§6.9). The adequacy of both definitions will be vindicated by the ability to recover in the CCT key axioms of set theory and mereology (§7).

**Remark.**

*It follows from x≤y that both x and y are sums. This can be seen as follows. If x≤y, by definition there are yy such that SUM(y:yy) and x≺yy. Since SUM(y:yy), y is a sum. Since x≺yy and yy is an input to the sum constructor, x is summable. Only individuals are summable, so x is an individual, i.e. a given or a sum. This implies that x is a sum, since it follows from collapse that givens are sums*.

Note that (A31) is a natural generalization of, and hence stronger than, the claim that every given is a mereological atom (relative to the above definition of parthood). Define an atom to be an individual whose only part is itself:

**Definition 29.**

ATOM(x)↔ISSUM(x)∧∀y(y≤x→y=x)

The claim that every given z is an atom can be also formalized as:

$$\begin{array}{rl} & \text{G}\text{IVEN}(z)\;\wedge \;\forall x(x≺xx\to \text{G}\text{IVEN}(x))\;\wedge \;\text{S}\text{UM}(z:xx)\to \\ & \;\;\;z≺xx\;\wedge \;\forall x(x≺xx\to x=z). \end{array}$$

That is, if a given is the sum of some givens, then ‘it is those givens’, i.e. it is the only member of those givens. Thus, where this principle says that every *given* is a unique sum of givens, namely, the sum of itself, (A31) says that every mereological object, *whether a given or not*, is a unique sum of givens.

**Note.**

*Various generalizations will eventually be needed. First, we want to allow the givens to come equipped with a mereological structure. This will involve exchanging (A31) for some other axiom(s). It will also involve changing the definition of mereological sum. Second, we want to add ‘decomposers’, which construct new parts of already existing objects. The result is a broader conception of what can exist at successor stages*.

An important feature of the CCT is that it enables us to derive standard mereological axioms. More precisely, we can prove that the sums, ordered by ≤, form an atomistic General Extensional Mereology (AGEM) whose atoms are precisely the givens (see Appendix D.2).

### 6.11. Left and right constructors

The left and right constructors, in combination with the pair constructors, build pairs in a symmetrical way to other constructors. They do so without the need for order. At present, pairs are built in two stages using these types of constructor. We are currently working on replacing this approach with a simpler, direct construction process that treats pairs as derived objects.

The left and right constructors prepare objects to become inputs for the pair constructor. The general picture is the following. A pair has a left coordinate and a right coordinate. In the CCT, inputs to constructors are pluralities, which have unordered members. So the pair constructor needs some information to select the member of the input plurality that will become the left coordinate and the member that will become the right coordinate. We supply this information by constructing two kind of *sui generis* position objects: left objects and right objects.

The construction of left and right object is constrained in two ways. The first is the size of the input plurality:

(A32)

$$\begin{array}{rl} \forall xx\forall x((\text{L}\text{EFT}(x:xx)\; & \vee \;\text{R}\text{IGHT}(x:xx))\;\to \;\\ & \forall y\forall z(y≺xx\;\wedge z≺xx\;\to y=z)) \end{array}$$

*(Any input plurality to the left or right constructor has no more than one member.)*

Since pluralities are not empty, this axiom ensures that the left and right constructors apply only to singleton pluralities. As a result, the single member of the singleton plurality used as input becomes a left or right object.

The second constraint concerns the sole member of the input plurality: it must be ‘positionable’. We define this property as follows:

**Definition 30.**

$$\begin{array}{rl} & \text{P}\text{OSITIONABLE}(x)\;↔\;\\ & \;(\text{I}\text{S}\text{S}\text{ET}(x)\;\vee \;\text{I}\text{NDIVIDUAL}(x)\;\vee \;\text{I}\text{S}\text{P}\text{AIR}(x)\;\vee \;\text{T}\text{RACE}(x)) \end{array}$$

**Note.**

*It follows that position objects are not themselves positionable. This is a manifestation of their supporting role: position objects mark prior objects of interest as left or right so that they can be used, so marked, in the construction of a pair. The position objects themselves are not objects of interest for this construction. As a result, the left and right constructors cannot be iterated. For example, there is no ‘left left object’*.

Once we have defined what counts as ‘positionable’, we sanction the second requirement by means of an axiom.

(A33)

$$\begin{array}{rl} \forall xx\forall x((\text{L}\text{EFT}(x:xx)\; & \vee \;\text{R}\text{IGHT}(x:xx))\;\to \;\\ & \forall z(z≺xx\;\to \text{P}\text{OSITIONABLE}(z))) \end{array}$$

*(If some things construct a left or right object, then each of them is positionable.)*

Every appropriate singleton plurality is eventually used to construct a left object and a right object:

(A34)

$$\begin{array}{rl} & \forall xx(\forall y\forall z(y≺xx\;\wedge \;z≺xx\;\to \;y=z)\;\wedge \;\\ & \;\forall z(z≺xx\to \;\text{P}\text{OSITIONABLE}(z))\to \\ & \;\;\;\exists x_{1}\text{L}\text{EFT}(x_{1}:xx)\;\wedge \;\exists x_{2}\text{R}\text{IGHT}(x_{2}:xx)) \end{array}$$

*(If xx is singleton plurality whose member is positionable, then a left object and a right object with input xx exist.)*

**Remark.**

*As remarked in §6.9, one can reason from the axioms of our theory to the conclusion that the elements of a set exist at an earlier stage than the set itself. An analogous reasoning yields that the sole member of the plurality from which a position object is constructed exists at an earlier stage than the position object itself. Formally, we have:*

∀x(x@t∧(LEFT(x:xx)∨RIGHT(x:xx))→∃s(s⊲t∧xx@@s)).

Like sets, position objects strictly depend on their input pluralities. So the left and right constructors are injective:

(A35) $\text{I}\text{NJECTIVE}(c_{\text{left}})\;\wedge \;\text{I}\text{NJECTIVE}(c_{\text{right}})$

*(Position types are injective. That is, a position object, left or right, is constructed from at most one plurality.)*

In combination with the functionality of the generic constructor, this axiom entails that position objects are extensional: two position objects of the same type are identical if and only if they are constructed from the same plurality.

### 6.12. Pair constructor

Our current approach to pairs avoids an explicit order, relying on left objects and right objects to mark positions in a pair. We recover the paired objects from the inputs that generated the position objects used to construct the pair. For example, suppose we want to pair a and b in this order, choosing left and right to represent the first and second coordinate, respectively. Then, we use the singleton plurality of a to construct a left object $a^{\prime }$, and we use the singleton plurality of b to construct a right object $b^{\prime }$. Finally, we build a pair from the plurality of $a^{\prime }$ and $b^{\prime }$. We recover the first coordinate by considering the sole member of the plurality that constructed the left object $a^{\prime }$. This yields a, as desired. We recover the second coordinate similarly.

This approach requires that the input plurality to the pair constructor have exactly two members: a left object and a right object. We formalize the requirement using the following axiom:

(A36)

$$\begin{array}{rl} \forall xx & \forall x(\text{P}\text{AIR}(x:xx)\;\;\to \;\\ & \exists y\exists z(y≺xx\;\wedge \;\text{I}\text{S}\text{L}\text{EFT}(y)\;\wedge z≺xx\;\wedge \;\text{I}\text{S}\text{R}\text{IGHT}(z)\;\wedge \\ & \forall w(w≺xx\;\to \;(w=y\;\vee \;y=z)))) \end{array}$$

*(Any input plurality to the pair constructor has exactly two members: a left object and a right object.)*

Thus, ‘pairable’ objects are left and right objects. As noted, however, not every plurality of pairable objects can be an input to the pair constructor. Pairable objects must combine in the particular way specified by the above axiom: exactly one left object and one right object.

There are additional axioms. Every appropriate plurality is eventually used to construct a pair:

(A37)

$$\begin{array}{rl} \text{I}\text{S}\text{L}\text{EFT}(x)\;\wedge & \;\text{I}\text{S}\text{R}\text{IGHT}(y)\to \\ & \exists zz(\forall z(z≺zz↔(z=x\vee z=y))\;\wedge \;\\ & \exists z\text{P}\text{AIR}(z:zz)) \end{array}$$

*(For every left object x and right object y, a pair with input x and y exists.)*

**Remark.**

*In analogy with the case of sets and position objects, we can prove from the axioms of the CCT that the objects used to build a pair exist at earlier stages than the pair itself. The claim is formalized as follows:*

PAIR(z:xx)∧z@t→∀x(x≺xx→∃s(s⊲t∧x@s)).

Moreover, like sets and position objects, pairs strictly depend on their input pluralities. So their constructor is injective:

(A38) $\text{I}\text{NJECTIVE}(c_{\text{pair}})$

*(The type pair is injective. That is, a pair is constructed from at most one plurality.)*

As before, the functionality of the generic constructor and the injectivity of the specific constructor entail extensionality. Here, we have that two pairs are identical if and only if they are constructed from the same plurality.

**Note.**

*In general, the present approach to pairs requires two stages to build a pair. We start with some objects a and b. At a subsequent stage, we turn them into position objects. It follow from (A35) that these position objects are distinct if a and b are distinct. The position objects are then used as inputs to the pair constructor at a later stage. There is some analogy with the construction of pairs as set-theoretical objects according to Kuratowski's definition. We start with a and b. We then build {a} and {a,b}. Finally, we build the desired set {{a},{a,b}}, which stands for the pair ⟨a,b⟩*.

**Note.**

*How can our approach be extended from pairs to tuples of arbitrary length? A natural option is to add a new constructor for each coordinate of the desired tuples. This would allow us to construct position objects corresponding to the relevant coordinates. In our setting, the effect of new constructors can be obtained by specializing the generic constructor through parameters. For these parameters, one could use, for example, set-theoretic renderings of the natural numbers associated with the target coordinates. So the number one (say {a} for some conventionally chosen object a) would be used as the type of constructor for the first coordinate of a tuple, the number two ({{a}}) would be used as the type of constructor for the second coordinate and so on. Once the appropriate position objects have been built, we obtain a tuple by applying the tuple constructor to such objects. This idea can be extended to infinite sequences simply by using a larger stock of parameters (e.g. a representation of the ordinals)*.

*An alternative option is to develop a general theory of sequences, perhaps based on two constructors: one constructor for turning an object into the corresponding sequence of length one and another for concatenating two sequences. We leave this for future work*.

### 6.13. Union constructor

Among the constructors countenanced by the CCT, the union constructor has a special status. It is the only *derived* constructor, in the following sense. The objects constructed by the union constructor are sets, which can also be constructed using the set constructor. By contrast, not all sets can be generated by means of the union constructor alone. For example, singleton sets of givens cannot be so constructed. Singleton sets are effectively ‘givens’ for the union constructor. The upshot is that the union constructor could be dispensed with. However, pragmatic concerns as well as faithfulness to the target TLOs recommend that the union constructor be included alongside the other constructors.

A set z is the union of the sets xx if and only if every member of xx is a set and something is an element of z just in case it is an element of some set in xx. So the union of the sets xx is the set constructed from the plurality of elements of members of xx. We capture the relation between xx and their members by means of a definition:

**Definition 31.**

$$\begin{array}{rl} \text{U}\text{NIONISE}(xx,yy)\; & ↔\;\\ & \forall y(y≺yy\;\to \;\text{I}\text{S}\text{S}\text{ET}(y))\;\wedge \;\\ & \forall x(x≺xx↔\exists y(y≺yy\;\wedge \;\exists zz(x≺zz\;\wedge \;\text{S}\text{ET}(y:zz)))) \end{array}$$

That is, UNIONISE(xx,yy) holds if and only if yy are some sets whose elements, combined, are all and only xx.

This relation between pluralities is used in the following axiom, which describes the link between the union constructor and the set constructor:

(A39) UNION(z:xx)↔∃yy(SET(z:yy)∧UNIONISE(yy,xx))

*(z is the union of xx if and only if z is the set of some yy that ‘unionise’ xx.)*

A consequence of the axioms is that if UNION(z:xx), then, each member of xx is a set. So ‘unionable’ objects are sets.

**Remark.**

*Since unions are sets, their identity conditions are inherited from those of sets and need not be specified separately. However, it may be useful to describe an appropriate identity criterion that could be adopted in contexts where the union constructor is not treated as derived:*

$$\begin{array}{rl} & \text{U}\text{NION}(z_{1}:xx_{1})\wedge \text{U}\text{NION}(z_{2}:xx_{2})\to \\ & \;(\exists yy(\text{U}\text{NIONISE}(yy,xx_{1})\wedge \text{U}\text{NIONISE}(yy,xx_{2}))↔z_{1}=z_{2}) \end{array}$$

*(The unions constructed from $xx_{1}$ and $xx_{2}$ are identical if and only if the same things unionise both $xx_{1}$ and $xx_{2}$.)*

**Remark.**

*We want to think of a union as dependent on the sets from which it is constructed. Nevertheless, we do not add an axiom to that effect. Once we identify unions with sets, a union exists at a stage if and only if the set to which it is identical exists at that stage. This means that a union can exist before some sets that could construct it exist. For example, if we have two givens a and b, we could form the set {a,b} before forming the singletons {a} and {b}. So the union of these singletons can exist before the singletons do*.

*Given our set-up, however, we can let traces express the idea that a union is generated after the sets that construct it. Consider the union z constructed from the sets xx. We introduce traces of the construction of z from xx at the first stage t such that z exists at t and xx exist at a stage prior to t. This simulates that z has been constructed from xx at t. Returning to our example, we record the construction of {a,b} as the union of {a} and {b} at the first stage where {a,b} exists after {a} and {b} have been constructed*.

### 6.14. Traces

The next set of axioms concerns traces. These axioms ensure that whenever certain constructions are effected, there are objects that encode relevant information about the construction process. The name ‘traces’ emphasizes that the role of these objects is to log information about constructions. Let us consider an example. Suppose the set z is constructed from xx, i.e. SET(z:xx). Then, for *each member* x of the input plurality xx, there is a trace w that stores the following information about the underlying construction process:

— the output (the object constructed),
— the fact that x is one of the inputs, and
— the type (set).

Using our primitive predicates, the information about the example would be formalized as follows:

— HASOUTPUT(w,z),
— HASINPUT(w,x), and
— $\text{H}\text{AS}\text{T}\text{YPE}(w,c_{\text{set}})$.

Thus, a trace can be seen as a reification of the constructional relation between composite and component, i.e. between the output and each member of the input to the construction. Suppose that we use the plurality of a and b, two distinct objects, to build the set z. Then two traces $w_{1}$ and $w_{2}$ emerge from this construction. Both traces have output z and type $c_{\text{set}}$. However, while $w_{1}$ has input a, $w_{2}$ has input b. In general, the information that z is a set constructed from some objects xx can be obtained by consulting all the traces that have the set z as an output.

We characterize the traces by specifying the behaviour of the primitive predicates associated with them. First, we require that an object always carries the threefold information just described or does not carry any information of this kind:

(A40)

$$\begin{array}{rl} & \forall w((\exists z\text{H}\text{AS}\text{O}\text{UTPUT}(w,z)\;↔\;\exists x\text{H}\text{AS}\text{I}\text{NPUT}(w,x))\;\wedge \\ & \;\;(\exists z\text{H}\text{AS}\text{O}\text{UTPUT}(w,z)\;↔\;\exists y\text{H}\text{AS}\text{T}\text{YPE}(w,y))) \end{array}$$

*(Any object has an output if and only if it has an input if and only if it has a type.)*

Thus, we can define a trace simply as an object that carries information about an output:

**Definition 32.**

TRACE(w)↔∃zHASOUTPUT(w,z)

We now lay out the identity conditions for traces:

(A41)

$$\begin{array}{rl} & \text{T}\text{RACE}(w_{1})\;\wedge \;\text{T}\text{RACE}(w_{2})\;\to \;\\ & \forall x_{1}\forall x_{2}\forall y_{1}\forall y_{2}\forall z_{1}\forall z_{2}((\text{H}\text{AS}\text{O}\text{UTPUT}(w_{1},z_{1})\;\wedge \;\text{H}\text{AS}\text{O}\text{UTPUT}(w_{2},z_{2})\;\wedge \;\\ & \;\text{H}\text{AS}\text{I}\text{NPUT}(w_{1},x_{1})\;\wedge \;\text{H}\text{AS}\text{I}\text{NPUT}(w_{2},x_{2})\;\wedge \;\\ & \;\text{H}\text{AS}\text{T}\text{YPE}(w_{1},y_{1})\;\wedge \;\text{H}\text{AS}\text{T}\text{YPE}(w_{2},y_{2}))\;\to \;\\ & \;\;(z_{1}=z_{2}\;\wedge \;x_{1}=x_{2}\;\wedge \;y_{1}=y_{2}\;↔\;w_{1}=w_{2})) \end{array}$$

*(Two traces are identical if and only if they have the same output, input and type.)*

**Remark.**

*Together with (A40), these identity conditions entail that any trace carries information about exactly one output, exactly one input, and exactly one type*.

The next step is to ensure that traces are introduced at the correct stages. That is, when an object is constructed at a stage—or, as in the case of union, it is *treated* as constructed at a stage—the appropriate traces are also introduced at that stage.

(A42)

$$\begin{array}{rl} & (\text{C}\text{ONSTRUCT}(z:xx,y)\;\wedge \;z@t\;\wedge \;\exists s(s⊲t\;\wedge \;xx@@s))\to \\ & \;\forall x(x≺xx\;\to \;\exists w(w@t\;\wedge \;\\ & \;\;\text{H}\text{AS}\text{O}\text{UTPUT}(w,z)\;\wedge \;\text{H}\text{AS}\text{I}\text{NPUT}(w,x)\;\wedge \;\text{H}\text{AS}\text{T}\text{YPE}(w,y))) \end{array}$$

*(Suppose z is constructed from xx with type y, z exists at t and xx exist at some stage before t. Then, we also require at t the existence of appropriate traces. That is, for every member x of xx, there is at t a trace recording that the construction process has output z, input x and type y.)*

**Remark.**

*The condition*

∃s(s⊲t∧xx@@s)

*in the antecedent of (A42) is redundant for sets, position objects and pairs. This is because, as remarked above, constructions of these types require the members of the input plurality to exist at an earlier stage than the constructed object. In the case of sums or unions, however, the condition is not redundant*.

*Some examples might help illustrate the effect of this condition. Let us use ‘+’ to indicate the result of summing some objects. And let us indicate the plurality of $x_{1},...,x_{n}$ as $\left[x_{1},...,x_{n}\right]$. So, for example, SUM(a+b:[a,b]). Suppose that the initial stage, stage 0, comprises three givens a, b and c. Suppose further that, at stage 1, we have the following individuals: a, b, c, a+b, a+c, b+c and a+b+c. Consider the construction SUM(a+b+c:[a+b,c]). While a+b and c exist at stage 1, no traces of this construction is introduced at stage 1. This is because a+b does not exist at the previous stage. The traces of SUM(a+b+c:[a+b,c]) are introduced at stage 2, when the conditions laid out in the antecedent of (A42) are met. By contrast, the traces of SUM(a+b+c:[a,b,c]) exist at stage 1, since a+b+c exists at that stage and each member of [a,b,c] exists at the previous stage. This distribution of traces expresses that the construction SUM(a+b+c:[a+b,c]) takes place at stage 2, while the construction SUM(a+b+c:[a,b,c]) takes place at stage 1*.

*Something similar applies in the case of unions. Suppose the only set constructed at stage 1 is {a,b,c}, whereas {a,b} and {c} are constructed at stage 2. While {a,b,c} is the union of {a,b} and {c}, it exists prior to them. In this scenario, the traces of UNION({a,b,c}:[{a,b},{c}]) are introduced at stage 3, namely, the first stage such that {a,b,c} exists at it and both {a,b} and {c} exist prior to it. This expresses that the construction UNION({a,b,c}:[{a,b},{c}]) takes place at stage 3*.

We have given sufficient conditions for the existence of traces, ensuring that construction processes are accompanied by the appropriate traces. We now want to give necessary conditions, ensuring that there are no more traces than required. This is done by an axiom stating that the only traces existing at a stage are those recording constructions (basic or derived) using objects from previous stages:

(A43)

$$\begin{array}{rl} & \text{T}\text{RACE}(w)\wedge w@t\to \\ & \;\exists z\exists xx\exists x\exists y\exists s(\text{C}\text{ONSTRUCT}(z:xx,y)\wedge z@t\wedge xx@@s\wedge s⊲t\;\wedge \\ & \;\;x≺xx\wedge \text{H}\text{AS}\text{O}\text{UTPUT}(w,z)\wedge \text{H}\text{AS}\text{I}\text{NPUT}(w,x)\;\wedge \;\text{H}\text{AS}\text{T}\text{YPE}(w,y)) \end{array}$$

*(If a trace w exists at a stage t, then it must correctly record some type of construction of an object existing at t from an input existing at some stage before t.)*

**Remark.**

*Axiom (A43) implies that there are six kinds of traces, corresponding to the five basic constructors (set, sum, left, right and pair) and to the derived constructor of union*.

### 6.15. Maximal extension of a stage

A stage t is a maximal extension of a stage s if and only if t contains every object that is constructed from objects that exist at s. This is formalized by the next definition:

**Definition 33.**

MAX(s,t)↔∀x(CONSTRFROM(x,s)→x@t)

**Remark.**

*The axioms for traces ensure that the appropriate traces exist at maximal stages*.

We assume maximality in the following sense:

(A44) ∀s∃tMAX(s,t)

*(Every stage has a maximal extension.)*

Given any stage, the axioms for the specific constructors ensure that the objects existing at that stage are used for every applicable construction. For example, axiom (A24) states that for every plurality xx of settable objects existing at a stage, the set of xx is constructed. Maximality requires the objects constructed from a given stage to exist together at some stage.

**Remark.**

*A maximal extension strictly succeeds the stage whose maximal extension it is:* MAX(s,t)→s⊲t.

**Note.**

*The theory implies that no new sums are constructed after the first maximal extension of the initial stage. This is because, as observed in §6.10, two sums are identical if and only if they are sums of the very same pluralities of givens. So once every plurality of givens has been summed, every sum that is constructed must be identical to one that has already been constructed*.

**Remark.**

*Consider the statement that every successor stage is also a maximal extension:*

(*)SUCC(s,t)→MAX(s,t).

This statement requires, in effect, that all constructional possibilities admitted by the theory be realized as soon as possible. Without the statement, the construction process may be slowed down: some constructional possibilities available at a stage maybe be realized before other constructional possibilities also available at that stage. For example, one may start by constructing mereological sums of givens before constructing sets of givens. Thus, (*) provides a switch that makes the construction process as fast as possible.

### 6.16. Induction on the construction of objects

The possibility of induction on the construction of objects is an appealing feature of the constructional approach, a feature that might not be available in a non-constructional setting. In §6.10, we used an induction on the construction of sums to show that every sum is a sum of givens. As noted, this specific form of induction relies essentially on the fact that givens and sums are individuals and that only individuals are summable. So, it has no obvious analogue in cases of constructors that also permit as inputs objects built from other constructors.

However, we have the resources to prove from our axioms a general form of induction on the construction of objects. Assume that

(i) every given is φ;
(ii) for every constructor, if the inputs are φ, the constructed object and its associated traces are φ.

Then, everything that is not a stage is φ.

We prove this as follows. Assume, for *reductio ad absurdum*, that the assumptions hold but there is a non-φ that is not a stage. By the well-foundedness of the stages, there is a least stage, s, at which such an object x exists. By the cumulativity of the stages, s cannot be a limit stage but must be a successor. By (A19) and (A43), x must either be constructed from some objects yy available at the preceding stage or be a trace associated to a construction effected at s. If x is constructed from yy, then each of yy is φ, since s is the least stage at which a non-φ exists. Then, it follows from assumption (ii) that x too is φ, contradicting our assumption that x is non-φ. If x is a trace, then, it emerges at s from a construction whose inputs are available at the preceding stage. Then, again, it follows from assumption (ii) that x too is φ, contrary to our assumption. We conclude that everything that is not a stage is φ. The proof appeals to stages and their well-foundedness, which highlights a benefit of the stage-theoretic framework.

## 7. Recovering set theory and mereology

As emphasized in §3, ontologies containing sets and sums usually adopt set theory and mereology as separate theories, without an integrated development. Our constructional approach permits a unified treatment of sets and sums. These target domains arise similarly through construction.

When set theory and mereology are adopted as separate theories, one relies on separate non-logical primitives: a relation of set-theoretic membership and a relation of parthood. In our setting, by contrast, there is no need for these new primitives. Both relations are defined in terms of more fundamental constructional notions, given in definitions 26 and 28 (see [11, pp. 567–568]). It might be useful to repeat the definitions. To be an element of a set is to be a member of the input plurality from which the set is constructed. To be a part of an object is to a member of a plurality from which the object can be built using the sum constructor. In short, to be an element or a part is to be capable of serving as input to the relevant construction.

We show these definitions to be adequate by demonstrating how to derive from them, in the CCT, the axioms of ZFC (minus the axiom of empty set) and those of atomistic AGEM. These immensely powerful axioms can thus be freely used within our constructional theory. A precise statement of the axioms recovered as well as some sample derivations can be found in Appendix D.

## 8. Consistency of the Core Constructional Theory

We can show that the CCT, axiomatized in §6, is consistent. As is often the case, this is proved by constructing a set-theoretic model in which every axiom of the theory is true. We offer some remarks about the actual model construction and the consistency proof, which are relatively simple.

To construct a set-theoretic model, we obviously need to rely on some set theory in the metalanguage. Suppose this set theory is X. We shall then have proved that ‘the CCT is consistent relative to X’, that is, if our set theory X is consistent, then so is the CCT. So it is important that X be some familiar set theory whose consistency is not disputed by set theorists. One such theory is ZFC. Our options concerning the choice of the theory X are either ZFC itself or some relatively minor variants.

— We can use a Morse–Kelley (MK) class theory, which adds to ZFC a single layer of classes on top of all the sets.
— We can use ZFC + an extra axiom stating that there exists an ‘inaccessible cardinal’. In the literature on strong axioms of infinity, this is considered a very modest extension of ZFC. (It is, however, stronger than MK, but has the advantage of talking only about sets, with no need to add a notion of class.)
— We can use ZFC as our meta-theory and prove the consistency of a slight weakening of the CCT obtained by restricting the axiom of plural comprehension. Instead of stating that any condition φ(x) that has instances can be used to define a plurality of all φs, we state that, for any given stage s, there is a plurality of all φs at stage s.
— We can drop the axiom of replacement from the CCT and use ZFC to prove the consistency of the resulting theory.

In short, the CCT can be proved to be consistent assuming some very modest extensions of standard set theory ZFC, and even the need to consider extensions can be avoided by weakening non-essential parts of the CCT. In Appendix E, we pursue the first option, namely, we show that the CCT is consistent relative to MK.

## 9. Conclusion

We set out to demonstrate the feasibility of a rigourous approach to constructional ontology. This has been established by producing a formalization of the CCO and by giving a mathematical proof of its consistency. This constructional approach to ontology unifies the core components of the target four-dimensionalist TLOs, leading to a single, common foundation for future development.

The formal theory, the CCT, unifies the three key domains—sets, sums and pairs—using a stage theory. In this theory, objects emerge through the application of different constructors to objects existing at the initial stage or constructed at prior stages. This unification and its associated simplification are apparent in the common development of the key domains, the common basis for the identity criteria of the objects in them, and the uniformity through which we capture other important commonalities and differences. We also demonstrate the strength of the CCT by recovering the axioms of standard set theory and mereology.

Future research might address and resolve some outstanding technical issues. For example, one might explore the introduction of deconstructors to work along constructors, thus enabling the possibility of mereological gunk. One might also explore alternative formalizations of the constructional approach, such as dynamic formalizations and formalizations that eliminate stages in favour of their associated pluralities.

## Figures and Tables

**Table:** 

type	construction	identity criteria
sets	all sets are constructed by the set constructor	two sets are identical iff they are constructed from the same plurality (equivalently, iff they have the same elements)
individuals	individuals are either constructed by the sum constructor or are givens	two individuals are identical iff they are constructed from the same givens (equivalently, iff they have the same parts)
left objects	all left objects are constructed by the left constructor	two left objects are identical iff they are constructed from the same plurality
right objects	all right objects are constructed by the right constructor	two right objects are identical iff they are constructed from the same plurality
pairs	all pairs are constructed by the pair constructor	two pairs are identical iff they are constructed from the same position objects (i.e. from the same plurality of left object and right object)
unions	all unions are constructed by the derivative union constructor	two unions are identical iff they are identical as sets (i.e. the sets to which they are equal are identical)
traces	all constructions have associated traces	two traces are identical iff they have the same type and record the same construction output and input

**Table:** 

type	settable	summable	positionable	pairable	unionable
sets	**✓**	**✗**	**✓**	**✗**	**✓**
individuals	**✓**	**✓**	**✓**	**✗**	**✗**
position	**✗**	**✗**	**✗**	**✓**	**✗**
objects					
pairs	**✓**	**✗**	**✓**	**✗**	**✗**
unions	**✓**	**✗**	**✓**	**✗**	**✓**
traces	**✓**	**✗**	**✓**	**✗**	**✗**

**Table:** 

CCT	BORO	IDEAS	ISO 15926	HQDM
‘Object’	objects	thing	thing	thing
individual	elements	individual	possible_individual	possible_individual
IsSet	types	type	class	class
IsPair	tuples	tuple	relationship	relationship
trace & $c_{\text{sum}}$	wholes-parts	wholePart	composition_of_individual	composition_of_individual
trace & $c_{\text{set}}$	types-instances	typeInstance	classification	classification
trace & $c_{\text{union}}$	super-sub-types	superSubType	specialization	specialization
‘Position object’	tuple-places	tuplePlaces	end	end

## Data Availability

This article has no additional data.

## Appendix A. Notions

### A.1. List of key types and identity criteria

The table below provides an informal summary of the seven broad types of objects in the CCT together with their identity criteria. There are six types of objects obtained from the application of constructors: sets, individuals, left objects, right objects, pairs and unions. In addition, there are traces, which emerge from the constructional process. As is clear from the description in the table, all these types of objects satisfy extensional identity criteria, which directly correspond to the identity criteria in the target TLOs (see Appendix C).

### A.2. Constraints on inputs

Constructional processes are constrained in that not all kinds of pluralities can serve as inputs to constructions. Moreover, different constructors may place different restrictions on their inputs. The following table displays these constraints based on the broad types of members in the input plurality. For example, the set constructor admits as inputs only pluralities whose members are sets, individuals, pairs, unions or traces. It is worth emphasizing that there is no requirement that such pluralities be uniform: a plurality is admissible as long as each of its members is of an admissible type. So one can form the set {a,w}, where a is an individual and w is a trace.

There are further constraints. Only singleton pluralities can be inputs to the construction of position objects. Moreover, the construction of a pair requires an input plurality containing a left object, a right object and nothing else. As explained in §6.12, the paired objects are ultimately recovered from the inputs that generated the position objects used to construct the pair. So, while only position objects are pairable, the intended coordinates of pairs are, in effect, the positionable objects: sets, individuals, pairs, unions and traces. Position objects function as mere intermediaries.

## Appendix B. Design choices

The present work aims to show the viability of a rigourous foundation for constructional ontology. We accepted that, as a reasonable cost for proof of concept, this would involve some compromises in the design choices.

We needed to identify a safe approach, in particular with respect to the type of formal framework used. The choice boiled down to stage theory or procedural postulationism (a theory outlined in [27]). While the latter is arguably more attuned to constructionalism, it would require the development of significant logical machinery. Accordingly, the safer option of stage theory, which has a better understood semantics, was chosen.

To appreciate some of our design choices, it might be useful to compare the CCT to the informal approach of [7–9], which we may call the *benchmark approach*. For sets, the formalization required no compromise; in this respect, the CCT realizes the benchmark approach. In other respects, there is some divergence.

The benchmark approach adopts a mixture of composing and decomposing constructors. Specifically, it adopts a decomposer to construct individuals. The construction process starts with the pluriverse as a single given. All individuals are then obtained by applying a sum decomposer to the pluriverse. By contrast, the CCT uses a single form of constructor (composers) across the board, thus avoiding the complications of managing both composers and decomposers. This meant we had to adopt a different approach to individuals, one that relies on composers. We chose to start with mereological atoms as givens (informally, all the mereological atoms in the pluriverse) and to use a sum composer to construct the rest of the individuals. In effect, this amounted to swapping a sum decomposer for a sum composer. The resulting ontology is therefore as granular as the givens. It does not provide ‘access’ to any parts that such givens might have or to whole-part relationships between the givens.

To build tuples, the benchmark approach uses a tuple constructor, assuming that this constructor can be applied to objects in a way that respects order and repetition. For this initial version of the CCT, we adopted a simpler approach that avoided the requirements for a formalization of order and repetition. This is a natural and familiar approach to n-tuples: it starts with primitive pairs and opens up the possibility of coding n-tuples through nesting.

Four general objects are included in the benchmark approach and the target TLOs: set type, set-member type, super-sub-set type and whole-part type. The decision has been made to incorporate them into the CCT as givens represented by special constants. As a result, these general objects can be inputs to constructors.

Given a constructional ontology, one might consider subontologies obtained from various possible selections of givens and constructors [10]. Investigating and comparing these subontologies might be useful. Partridge *et al*. [7] have found that it helps expose and explain the metaphysical structures of the candidate ontologies, and that it helps assess different architectural choices. The benchmark approach made use of ‘ontological sandboxes’ to allow the development and study of subontologies in isolation from the full constructional ontology. There was no attempt to formalize this feature in the CCT. However, the CCT has distinctive characteristics whose explanatory benefits resemble those of ontological sandboxes. The CCT allows non-maximal stages, and it could be modified to allow multiple initial stages, each with its own givens. This allows for varieties of ontogeneses where constructors are exhausted in independent branches before merging—mimicking the behaviour of ontological sandboxes. For example, one could exhaust the sum constructor, constructing all individuals, before starting to apply the other constructors.

One question that emerges clearly in the formalization is how far the construction process should go and, in particular, whether it should be extended into the transfinite. There is a requirement for the transfinite in the target TLOs; hence, we have included it in the CCT.

## Appendix C. Supporting the target top-level ontologies

The CCO, and so the CCT, is intended to be the foundation of the IMF's TLO. This was a key part of the planned process of developing an FDM seed from a set of four-dimensionalist TLOs that best meet its technical requirements [4]. The table below shows how the CCO maps directly onto, and thereby supports, these selected TLOs.

Note that the CCO has a general trace whose type can be recovered, whereas the other TLOs have individual types for each trace.

## Appendix D. Derivation of set theory and mereology

### D.1. Axioms of set theory

This section presents key set-theoretic axioms that can be recovered within the CCT. Our exposition of the axioms follows [25, §4.7]. Standard set theory, ZFC, is a theory of pure sets, formulated in the language of first-order logic containing only one non-logical predicate, ∈ for membership. (All other set-theoretic notions are defined in terms of this single predicate.) The CCT describes, in addition, entities that are not sets. So the appropriate set-theoretic axioms in our context make room for such entities. This can be done by suitable restrictions of the quantifiers in the axioms of ZFC. The result is similar to what is often known as ZFCU, namely, ZFC with *urelements*. The traditional role of urelements is played here by the givens alone, not by all entities that are not sets. We recognize various non-sets in addition to the givens. Indeed, some of these non-sets arise arbitrarily high in the hierarchy of stages, which implies that they do not form a set. By contrast, there is a set of all givens. This corresponds to the traditional axiom that the urelements form a set.

Another important difference between our target set-theoretic axioms and the axioms of ZFC concerns the empty set. ZFC requires the existence of an empty set, which is unavailable in the CCT. So we must also modify the axioms of ZFC to avoid any commitment to the empty set.

A third difference arises from the fact that not all objects in the CCT are settable. For example, position objects cannot be used to form sets. So, in formulating the target set-theoretic axioms, we must take care to respect this fact.

Below we list the target axioms. Then, we show how to derive them from the axioms of the CCT. Recall that we have provided a constructional definition of ∈ in §6.9: x∈y if and only if y is a set constructed from some plurality that has x as a member. This definition is operative in the following axioms.

(Extensionality) ISSET(x)∧ISSET(y)→(∀u(u∈x↔u∈y)→x=y)
*(Coextensive sets are identical.)*

(Pairing)

$$\begin{array}{rl} & \text{S}\text{ETTABLE}(x)\;\wedge \text{S}\text{ETTABLE}(y)\;\to \\ & \;\;\exists z(\text{I}\text{S}\text{S}\text{ET}(z)\;\wedge \;\forall u(u\in z↔u=x\vee u=y)) \end{array}$$

*(Every two settable objects have a pair set.)*

(Union)

$$\begin{array}{rl} & \text{I}\text{S}\text{S}\text{ET}(x)\;\wedge \forall y(y\in x\;\to \text{I}\text{S}\text{S}\text{ET}(y))\;\to \\ & \;\exists z(\text{I}\text{S}\text{S}\text{ET}(z)\;\wedge \;\forall u(u\in z↔\exists w(u\in w\wedge w\in x)) \end{array}$$

*(For every set x whose elements are sets, there is a set y whose elements are precisely those objects that are elements of some element of x.)*

(Powerset)

$$\begin{array}{rl} & \text{I}\text{S}\text{S}\text{ET}(x)\;\to \exists y(\text{I}\text{S}\text{S}\text{ET}(y)\;\wedge \;\\ & \;\;\;\;\forall u(u\in y↔\text{I}\text{S}\text{S}\text{ET}(u)\wedge \forall w(w\in u\;\to \;w\in x)) \end{array}$$

*(Every set has a powerset.)*

(Infinity)

$$\begin{array}{rl} & \exists x\exists y(\text{G}\text{IVEN}(y)\;\wedge \;y\in x\;\wedge \;\forall z(z\in x\;\to \\ & \;\;\;\exists u(\forall w(w\in u\;↔\;w=z)\;\wedge \;u\in x))) \end{array}$$

*(There is an infinite set. More specifically, there is a set x with a given as an element and such that, whenever z is an element of x, so too is its singleton {z}.)*

(Separation)

$$\begin{array}{r} \exists y(y\in x\;\wedge \;\varphi (y))\to \exists z\forall u(u\in z↔u\in x\;\wedge \;\varphi (u)) \end{array}$$

*(For any set x and any condition φ satisfied by at least one element of x, there is a set of precisely those elements of x that satisfy φ.)*

(Foundation)

$$\begin{array}{r} \text{I}\text{S}\text{S}\text{ET}(x)\;\to \;\exists y(y\in x\wedge \neg \exists z(z\in y\wedge \;z\in x)) \end{array}$$

*(Every set x has an element that is disjoint from x.)*

(Replacement)

$$\begin{array}{rl} & \text{I}\text{S}\text{S}\text{ET}(x)\;\wedge \;\forall y\forall z(\psi (y,z)\;\to \;\text{I}\text{S}\text{S}\text{ETTABLE}(z)\wedge \forall u(\psi (y,u)\;\to \;u=z))\;\wedge \;\\ & \;\exists y\exists z(y\in x\;\wedge \psi (y,z))\;\to \\ & \;\;\exists w\forall u(u\in w\;↔\;\exists y(y\in x\;\wedge \;\psi (y,u))) \end{array}$$

*(For every set x and functional condition ψ whose image includes only settable objects, if some element of x bears ψ to some object, then there is a set of precisely those objects that are borne ψ by some element of x.)*

This axiom is based on a simple and intuitive idea. Consider any set. For each of its elements, choose either to keep this element or to replace it with some other settable object. Then the resulting collection is also a set.

(Choice)

$$\begin{array}{rl} & \text{I}\text{S}\text{S}\text{ET}(x)\;\wedge \;\forall y(y\in x\;\to \;\text{I}\text{S}\text{S}\text{ET}(y))\;\wedge \;\\ & \;\forall y\forall z(y\in x\;\wedge \;z\in x\;\wedge \;y\neq z\;\to \;\neg \exists u(u\in y\;\wedge \;u\in z))\;\to \;\\ & \;\;\exists w(\text{I}\text{S}\text{S}\text{ET}(w)\;\wedge \;\forall y(y\in x\;\to \\ & \;\;\;\exists u_{1}(u_{1}\in y\;\wedge \;u_{1}\in w\;\wedge \forall u_{2}(u_{2}\in y\;\wedge \;u_{2}\in w\;\to u_{2}=u_{1})))) \end{array}$$

*(Every set x of non-empty disjoint sets has a choice set, that is, a set containing precisely one element of each element of x.)*

An example owing to Russell might be useful to understand the Axiom of Choice. Suppose you have infinitely many pairs of shoes. Then, it is easy to define a set containing precisely one member of each pair, namely, the set of left shoes. What if you have infinitely many pairs of socks where the two members of each pair are indistinguishable? Then we are unable to define a set containing precisely one member of each pair. The Axiom of Choice tells us that such a set exists, irrespective of our ability to define it.

It is fairly straightforward to prove that the axioms of set theory just stated can be recovered in our theory. That is, they can be derived from the axioms and definitions of the CCT. The derivation relies essentially on the principles of PFO+ embedded in the theory. To illustrate this, we describe two cases: pairing and powerset.

*Pairing*. We want to show that every two settable objects have a pair set. Consider two settable objects x and y. Since stages are cumulative, there is a stage at which each of the two objects exists. By plural comprehension, we can form the plurality of x and y, which exists at that stage. Thus, by (A24), there is the set of x and y, which is our desired pair set.

*Powerset*. We want to show that every set has a powerset. Let x be a set whose elements are yy. If x exists at a stage, yy also exist at that stage. It follows from (A24) that for every subplurality zz of yy, the set of zz exists. The maximality axiom (A44) yields a stage t at which every set constructed from a subplurality of yy exists. Consider the plurality ww of all such sets. Since this plurality exists at t, we can use (A24) to conclude that there is the set of ww, which is the desired powerset.

### D.2. Axioms of mereology

This section provides an exposition of the axioms of atomistic AGEM. Several equivalent axiomatizations of GEM can be found in the literature. The axioms adopted here are based on those proposed in [28] (see also [25, pp. 96–103]). We could simply import these axioms and ensure they have the appropriate scope by restricting all their quantifiers to sums. As remarked in §6.10, it follows from x≤y that both x and y are sums. This enables us to simplify some of the axioms by dropping redundant quantificational restrictions.

First, the sums are a partial order with respect to ≤:

(Reflexivity) ISSUM(x)→x≤x
*(≤, restricted to sums, is reflexive.)*

(Antisymmetry) x≤y∧x≤y→x=y)
*(≤, restricted to sums, is antisymmetric.)*

(Transitivity) x≤y∧y≤z→x≤z)
*(≤, restricted to sums, is transitive.)*

Next there is an axiom scheme of unrestricted fusion. For every formula φ, where x but neither y nor z occurs free, we have:

(Unrestricted Fusion)

$$\begin{array}{rl} \forall x(\varphi (x) & \to \text{I}\text{S}\text{S}\text{UM}(x))\;\wedge \;\exists x\varphi (x)\to \\ & \exists y(\text{I}\text{S}\text{S}\text{UM}(y)\wedge \forall z(y\leq z↔\forall x(\varphi (x)\to x\leq z))) \end{array}$$

*(Suppose that every φ is a sum and there is at least one φ. Then, there is a sum y such that for every z, y≤z if and only if, for every x that is φ, x≤z. This means that there is a LUB, with respect to ≤, of the φs.)*

The last axiom of GEM is a principle of complementation (called ‘Remainder’ in [28]):

(Complementation)

$$\begin{array}{rl} \text{I}\text{S}\text{S}\text{UM}(x)\; & \wedge \;\text{I}\text{S}\text{S}\text{UM}(y)\;\wedge \;\neg x\leq y\;\to \\ & \exists z(\text{I}\text{S}\text{S}\text{UM}(z)\;\wedge \\ & \;\forall w(w\leq z↔(w\leq x\wedge \neg \exists u(\text{I}\text{S}\text{S}\text{UM}(u)\wedge u\leq w\wedge u\leq y)))) \end{array}$$

*(For any two sums x and y, if x is not part of y, there is a sum z whose parts are all and only the parts of x that do not overlap y. Two sums overlap if a sum is part of both. The sum z can be regarded as the relative complement of y with respect to x.)*

We obtain the target theory, AGEM, by adding an axiom of atomicity. Recall the definition of atom from §6.10:

ATOM(x)↔ISSUM(x)∧∀y(y≤x→y=x)

Then, the axiom of atomicity states:

(Atomicity) ISSUM(x)→∃y(ATOM(y)∧y≤x)
*(Every sum has an atomic sum as a part.)*

This completes the presentation of the axioms of AGEM.

As in the case of set theory, it is fairly straightforward to derive the axioms presented here from the CCT. The result is that, in the CCT, the sums ordered by ≤ satisfy the axioms of AGEM, where the atoms are precisely the givens. An important role in the derivation of the axioms is played by the principle that two sums are identical if and only if they are constructed from the same givens (§6.10). Particularly useful are (i) the result that every sum is a sum of givens (§6.10), and (ii) the following fact linking parthood with inclusion among pluralities of givens.

*Fact*: x≤y if and only if the givens whose sum is x are among the givens whose sum is y.

*Proof*. We start with the right-to-left direction of the biconditional. Assume that x and y are the sums of gg and hh, respectively, where gg≼hh. Then by levelling, y is also the sum of the plurality zz obtained from hh by replacing gg with their sum. But the sum of gg is x. So x is one of some objects (i.e. zz) whose sum is y. This means that x≤y. We now show the left-to-right direction. Assume that x≤y, that is, x is one of some objects yy whose sum is y. Let g be one of the givens whose sum is x. Then, by levelling, x is also the sum of x and g. Let zz be the plurality obtained from yy by replacing x with x and g. By levelling again, the sum of zz is y. Since g is one zz, it follows that g is one of the givens whose sum is y. Since g was chosen arbitrarily from the givens whose sum is x, we conclude that the givens whose sum is x are among the givens whose sum is y.

As before, we give two examples, showing how to recover Antisymmetry and Unrestricted Fusion.

*Antisymmetry*. Assume that x≤y and y≤x. We want to show that x=y. Suppose x and y are the sums of givens gg and hh, respectively. By the Fact, we have that gg≼hh and hh≼gg, which entails that gg≈hh. It follows from the functionality of the generic constructor that x=y. *Unrestricted Fusion*. Assume that every φ is a sum and there is at least one φ. We want to show that there is a LUB, with respect to ≤, of the φs. Since there is some φ, plural comprehension yields the givens that are part of some φ. By (A28), their sum y exists. Clearly, y is an upper bound of the φs. To show that y is the LUB, suppose z is another upper bound. Since every x satisfying φ is part of z, we must also have, by the transitivity of ≤ (established above), that every given that is part of some φ is part of z. Thus, every given from which y is constructed is part of z. Moreover, from the fact that x≤y if and only if the givens whose sum is x are among the givens whose sum is y, it follows that every given from which y is constructed is also one of the givens from which z is constructed. It now follows, again from that fact, that y≤z and thus also that y is the LUB, as desired.

## Appendix E. Proof of consistency of the Core Constructional Theory

We prove the consistency of the CCT by constructing a set-theoretic model M in which all the axioms of the theory are true. An informal description of the background semantics for the language of the CCT was given in §5.

For convenience, we work in set theory with urelements. Since our urelements form a set, it is routine to translate this impure set theory into a corresponding pure set theory. Our meta-theory will mostly be ZFC, but at times it will also be useful to invoke MK set theory, which adds to ZFC a single layer of classes on top of all the sets.

The model we construct validates the full CCT, including the optional additional principle (*) to the effect that every successor stage is a maximal extension (§6.15). In future work, it would be interesting to consider alternative, or more general, set-theoretic constructions which model other stage-theoretic axioms and assumptions.

As a basis for the model, we assume *two disjoint sorts of urelements*:

— A set I of index objects that encode the basic type of the objects that are modelled: **s** for set, **m** for (mereological) individual, **l** for left object, **r** for right object, **p** for pair and **t** for trace. (We do not need to include an index object for the derived type union.) That is, I={s,m,l,r,p,t}. We require that the index objects are not used in any other way in the set-theoretic model.
— A set G that will be used to build the objects representing the givens.

We also assume G has the following subset:

— A set W of the six constants indicating types of construction. So $W=\{c_{\text{set}},c_{\text{sum}},c_{\text{left}},c_{\text{right}},c_{\text{pair}},c_{\text{union}}\}$.

Except for stages, which will be treated separately, we model each object recognized by the CCT as a pair consisting of an index object and a set. The index object specifies what *kind* of object is being represented. The set specifies the particular object of this kind that is being represented. In characterizing the set-theoretic model, we adopt the usual notation for ordered tuples. The notation (a,b) stands for the Kuratowski pair, i.e. $\left(a,b){=}_{\text{def}}\{\{a\},\{a,b\}\right\}$. The notation (a,b,c) stands for ((a,b),c). And so on for longer tuples.

A remark about the role of the index objects: their function is to avoid clashes between types, or more precisely, to prevent confusion over which type of object is represented by a certain set, which would also lead to unclarity about *which object* is represented by the set in question. For example, without index objects, the set {{a},{a,b}} might be taken to represent either itself, i.e. a set of rank 2, or the pair of a and b.

It is useful to define the following notions of representation relative to a type. For any z in M, the domain of M:

— z *represents a set* x iff z=(s,x).
— z *represents an individual* iff z=(m,x), for some non-empty x⊆G. In this case, we also say that z *represents a mereological sum of* x.
— z *represents a left object* x iff z=(l,x).
— z *represents a right object* x iff z=(r,x).
— z *represents a pair* x iff z=(p,x).
— z *represents a trace* iff $z=(t,x_{1},x_{2},x_{3})$ and one of the following is the case:
  (i) $x_{3}=c_{\text{set}}$, $x_{1}$ represents a set y and $x_{2}$ is a member of y;
  (ii) $x_{3}=c_{\text{sum}}$, $x_{1}$ represents a mereological sum of y and $x_{2}$ represents a mereological sum of a subset of y;
  (iii) $x_{3}=c_{\text{left}}$, $x_{1}$ represents a left object y and $x_{2}$ is a member of y;
  (iv) $x_{3}=c_{\text{right}}$, $x_{1}$ represents a right object y and $x_{2}$ is a member of y;
  (v) $x_{1}=c_{\text{pair}}$, $x_{1}$ represents a pair y and $x_{2}$ is a member of y;
  (vi) $x_{1}=c_{\text{union}}$, $x_{1}$ represents a set y and $x_{2}$ represents a set $y^{\prime }\subseteq y$.

We say that an object in M is settable iff it represents a settable type (see Appendix A.2 for a list of settable types). Analogous definitions apply to summable and positionable objects.

To specify the domain of the model, we need to define a construction operation C that, given a set X, will simulate the result of undertaking all our forms of construction on the basis of the objects represented by elements of X. The operation will also simulate the introduction of the appropriate traces based on these constructions. That is, given a set X, we define C(X) as the set whose elements are all and only the following objects:

— for any non-empty x⊆X such that every element of x is settable, (s,x)∈C(X) and $\left(t,(s,x),y,c_{\text{set}})\in C(X\right)$ for every y∈x;
— for any non-empty x⊆X such that every member of x is summable, (m,z)∈C(X), where z is the union of all the sets that figure as second coordinates of elements of x and $\left(t,(m,z),y,c_{\text{sum}})\in C(X\right)$ for every y∈x;
— for any singleton x⊆X whose element y is positionable, (l,x)∈C(X) and $\left(t,(l,x),y,c_{\text{left}})\in C(X\right)$;
— for any singleton x⊆X whose element y is positionable, (r,x)∈C(X) and $\left(t,(r,x),y,c_{\text{right}})\in C(X\right)$;
— for any x⊆X with exactly two elements $y_{1}$ and $y_{2}$ representing a left object and a right object, (p,x)∈C(X), $\left(t,(p,x),y_{1},c_{\text{pair}})\in C(X\right)$ and $\left(t,(p,x),y_{2},c_{\text{pair}})\in C(X\right)$;
— for any non-empty x⊆X such that every element of x represents a set, $\left(t,(s,z),y,c_{\text{union}})\in C(X\right)$ for every y∈x, where z is the union of all the sets that figure as second coordinates of elements of x.

Let $G^{*}$ be {(m,{g}):g∈G}. The elements of $G^{*}$ represent the givens in the model. By transfinite recursion, we now define a sequence $M_{\alpha }$ such that

— $M_{0}=G^{*}$
— $M_{\alpha +1}=M_{\alpha }\cup C(M_{\alpha })$ and
— $M_{\lambda }=\underset{\gamma <\lambda }{⋃}M_{\gamma }$.

Exactly as we talk about the set-theoretic universe V as the union of all the ranks $V_{\alpha }$ (see [29, §§I.1 and II.10]), we can talk about the union of all the $M_{\alpha }$. We call this union $M^{-}$ to indicate that it is *almost* the domain M of our model. Here, it is convenient to use MK rather than ZFC as our meta-theory. Then, $M^{-}$ can be a proper class. Alternatively, if we use ZFC + ‘there is an inaccessible cardinal’, $M^{-}$ can be a set—more specifically, a subset of $M_{\kappa }$ for κ the first inaccessible.

Lemma 1.
*When* $(m,x)\in M^{-}$, then x⊆G.

*Proof*. A straightforward induction on the construction of $M^{-}$.

As noted, $M^{-}$ is almost the domain we need. To represent the stages, which do not emerge from constructions, we add to $M^{-}$ appropriate ordinals from our meta-theory. We assume that there is no overlap between $M^{-}$ and these ordinals. We set M to be the resulting domain. If we use MK, M is a proper class: the union of $M^{-}$ and the proper class of all ordinals. If we use ZFC + ‘there is an inaccessible cardinal’, M is a set: the union of $M^{-}$ and the set of ordinals less than the first inaccessible (relative to ≤, the canonical well-ordering of the ordinals).

The interpretation of the quantifiers of the CCT is determined by M. The first-order quantifiers range over elements of M. Plural quantifiers range over non-empty subclasses of M (if we use MK class theory) or non-empty subsets of M (if we use ZFC + ‘there is an inaccessible cardinal’). Plural membership is interpreted as set- or class-theoretic membership, i.e. ‘≺’ is true of a,b iff a∈b.

It remains to specify the interpretation i of terms and predicates of the CCT. The language includes six special constants, the elements of the set W, i.e. $\left\{c_{\text{set}},c_{\text{sum}},c_{\text{left}},c_{\text{right}},c_{\text{pair}},c_{\text{union}}\right\}$. For any constant c∈W, i(c) is the pair (m,c). These pairs are in M because W⊆G and $G^{*}=M_{0}$. The interpretation of the predicates is given by the following clauses:

— For every x, y and z, (x,y,z)∈i(CONSTRUCT) iff z=(m,c), where c∈W and:
  a. if $c=c_{\text{set}}$, y is a non-empty subset of settable elements of M and x=(s,y);
  b. if $c=c_{\text{sum}}$, y is a non-empty subset of summable elements of M, w is the union of all the sets that figure as second coordinates of elements of y and x=(m,w);
  c. if $c=c_{\text{left}}$, y is the singleton of a positionable element of M and x=(l,y);
  d. if $c=c_{\text{right}}$, y is the singleton of a positionable element of M and x=(r,y);
  e. if $c=c_{\text{pair}}$, $y=\{w_{1},w_{2}\}$, where $w_{1}$ is an element of M representing a left object, $w_{2}$ is an element of M representing a right object and x=(p,y);
  f. if $c=c_{\text{union}}$, y is a non-empty subset of M whose elements represent sets, u is the union of all sets that figure as second coordinates of elements of y and x=(s,y).
— For every x, x∈i(INDIVIDUAL) iff x∈M and x=(m,y), for some y.
— For every x and y, (x,y)∈i(HASOUTPUT) iff x∈M and $x=(t,y,x_{2},x_{3})$, for some $x_{2},x_{3}$.
— For every x and y, (x,y)∈HASINPUT iff x∈M and $x=(t,x_{1},y,x_{3})$, for some $x_{1},x_{3}$.
— For every x and y, (x,y)∈i(HASTYPE) iff x∈M and $x=(t,x_{1},x_{2},y)$, for some $x_{1},x_{2}$.
— For every α, α∈i(STAGE) iff α is an ordinal in M.
— For every α and β, (α,β)∈i(⊴) iff α and β are ordinals in M such that α≤β.
— For every x and α, (x,α)∈i(@) iff $x\in M_{\alpha }$ and α is an ordinal in M.

Finally, the interpretation of the vocabulary introduced by definition is determined by the interpretation of the defining expressions.

Theorem 1.
*All axioms of the CCT are true in* M.

*Proof*. We sketch the reasoning why the axioms satisfied in the model, proceeding by groups of axioms.

— The axioms of plural logic, including instances of comprehension, are satisfied because plural variables are interpreted as ranging over all non-empty subcollections of M. These subcollections will be either subclasses or subsets, depending on whether we use MK or ZFC + ‘there is an inaccessible’. (Notice that to validate comprehension axioms that give pluralities not bounded by a stage, we need to go beyond ZFC. This is the only place where that is needed.)
— The axioms concerning stages are satisfied because these axioms are true of the canonical ordering (α≤β) of the ordinals. Notice that the proofs that the stage-theoretic axioms of Infinity and Replacement are satisfied uses the corresponding axioms of set theory.
— The axioms concerning the initial stage are satisfied because $M_{0}$ has been chosen so as to be non-empty and to contain the interpretations of the six special constants.
— The axioms concerning what exists at a stage are satisfied by construction of $M^{-}$: every non-stage exists at a stage; the stages are cumulative; limit stages just accumulate the preceding stages; stages with identical domains are identical; and successor stages contain only the domain of the preceding stage, objects that can be constructed from this domain and appropriate traces.
— There are two axioms concerning the generic constructor. The definition of the construction operator C and the interpretation of the predicate CONSTRUCT ensures that objects of the same type, constructed from the same pluralities, are identical. Moreover, the interpretation has been constrained so that if an object is constructed from a plurality using a parameter, the parameter is a type of construction. So both axioms are satisfied.
— The classification axioms are satisfied by the way in which different types of objects have been encoded in the model. This encoding represents different types of constructed objects by means of different indices, except for unions, which are sets.
— The axioms concerning the set constructor are satisfied. The definition of the construction operator C implies that every plurality of settable objects existing at a stage (and no other plurality at that stage) forms a set at the next. Moreover, since the stages are well-founded, the elements of a set exist prior to the set. The extensionality of sets in the metalanguage secures the extensionality of sets in the object language, which means that the injectivity axiom is satisfied. The set-theoretic version of the axiom that there is an infinite stage holds at $M_{\omega }$ and is thus satisfied.
— The axioms concerning the sum constructor are satisfied because of the definition of the construction operator C and of the fact that the sums are tracked in our model by the unique set of givens from which they are constructed, which also implies that collapse and levelling are satisfied.
— The axioms concerning the left and right constructors are satisfied. The definition of the construction operator C implies that every singleton of a positionable object existing at a stage forms a position object at the next. Since the stages are well-founded, the element of a singleton of a positionable object exists prior to the resulting position object. Given our representation of position objects in the model, the extensionality of sets in the metalanguage secures the extensionality of position objects in the object language. This means that the injectivity axioms are satisfied.
— The axioms concerning the pair constructor are satisfied. The definition of the construction operator C implies that every set of exactly one left object and one right object existing at a stage forms a pair at the next stage. Since the stages are well-founded, the position objects exist prior to the pair. Given our representation of pairs in the model, the extensionality of sets in the metalanguage secures the extensionality of pairs in the object language. So the injectivity axiom is satisfied.
— Unlike other constructors, the union constructor is derived. Applications of this constructor yield sets rather than *sui generis* objects. The interpretation of the predicate for the generic constructor ensures that, when the construction type is union, the result is the appropriate set. So the single axiom concerning this constructor is satisfied.
— The axioms concerning traces are satisfied because the definition of the construction operator C ensures that all required traces are generated at the right stages. Together with each constructed object, C adds traces with the appropriate information about input, output and type of the construction.
— Finally, the model has been constructed so as to satisfy the axiom that every successor stage is maximal. This is because the construction operation C yields all our forms of construction from the objects at a given stage, and it generates all required traces.
